# Increased mitochondrial calcium uptake and concomitant mitochondrial activity by presenilin loss promotes mTORC1 signaling to drive neurodegeneration

**DOI:** 10.1111/acel.13472

**Published:** 2021-09-09

**Authors:** Kerry C. Ryan, Zahra Ashkavand, Shaarika Sarasija, Jocelyn T. Laboy, Rohan Samarakoon, Kenneth R. Norman

**Affiliations:** ^1^ Department of Regenerative and Cancer Cell Biology Albany Medical College Albany New York USA

**Keywords:** aging, Alzheimer, *Caenorhabditis elegans*, calcium, mitochondria, mTORC1, presenilin

## Abstract

Metabolic dysfunction and protein aggregation are common characteristics that occur in age‐related neurodegenerative disease. However, the mechanisms underlying these abnormalities remain poorly understood. We have found that mutations in the gene encoding presenilin in *Caenorhabditis elegans*, *sel*‐*12*, results in elevated mitochondrial activity that drives oxidative stress and neuronal dysfunction. Mutations in the human presenilin genes are the primary cause of familial Alzheimer's disease. Here, we demonstrate that loss of SEL‐12/presenilin results in the hyperactivation of the mTORC1 pathway. This hyperactivation is caused by elevated mitochondrial calcium influx and, likely, the associated increase in mitochondrial activity. Reducing mTORC1 activity improves proteostasis defects and neurodegenerative phenotypes associated with loss of SEL‐12 function. Consistent with high mTORC1 activity, we find that SEL‐12 loss reduces autophagosome formation, and this reduction is prevented by limiting mitochondrial calcium uptake. Moreover, the improvements of proteostasis and neuronal defects in *sel*‐*12* mutants due to mTORC1 inhibition require the induction of autophagy. These results indicate that mTORC1 hyperactivation exacerbates the defects in proteostasis and neuronal function in *sel*‐*12* mutants and demonstrate a critical role of presenilin in promoting neuronal health.

## Graphical abstract

Proteostasis decline is a common feature in age‐related disease. Loss of SEL‐12/presenilin function leads to increased calcium transfer from the endoplasmic reticulum to the mitochondria resulting in mitochondrial hyperactivity. The mitochondrial hyperactivity promotes mTORC1 signaling that deregulates proteostasis by inhibiting autophagy, which ultimately impairs neuronal fitness.

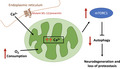

## 1 INTRODUCTION

The prevalence of neurodegenerative diseases is constantly increasing in the ever‐expanding elderly population. These diseases, such as Alzheimer's disease (AD), Huntington's disease, Lewy body dementia, and Parkinson's disease, all share a similar neuropathology that involves protein misfolding and aggregation. For example, AD is associated with protein aggregation in the form of amyloid plaques and neurofibrillary tangles. Biological processes that maintain protein homeostasis (proteostasis) decline with age and this decline is closely linked with neurodegenerative diseases (Kaushik & Cuervo, 2015; Labbadia & Morimoto, 2015; Yerbury et al., 2016). However, the mechanisms underlying this decline during aging and neurodegenerative disease progression are not clear.

Mutations in the presenilin encoding genes (*PSEN1* and *PSEN2*) are the primary cause of early‐onset, familial AD (FAD), but how presenilin dysfunction promotes neurodegeneration remains disputed. Presenilins are a highly conserved family of proteins that commonly reside on endomembranes such as the endoplasmic reticulum (ER) in organisms as diverse as plants to humans (Smolarkiewicz et al., 2013). Although presenilin 1 and 2 are primarily known to function as the catalytic component of the γ‐secretase complex, which is involved in the cleavage of the amyloid precursor protein (APP) to produce amyloid beta (Aβ) peptides, many studies have demonstrated a critical γ‐secretase independent role for presenilins in calcium homeostasis, mitochondrial and lysosome function and autophagy (Cheung et al., 2008; Lee et al., 2010; Neely et al., 2011; Nelson et al., 2007; Reddy et al., 2016; Sarasija et al., 2018; Tu et al., 2006). Importantly, many of these cellular processes are reported to be disrupted in AD and other neurodegenerative diseases (Orr & Oddo, 2013; Supnet & Bezprozvanny, 2011; Zare‐Shahabadi et al., 2015). Therefore, discovering the role of presenilin in these functions may help elucidate the underlying cause of AD and provide insight into how to treat neurodegenerative diseases.

Since presenilin proteins are highly conserved, we have turned to the invertebrate model system *Caenorhabditis elegans* to help identify the role presenilin may have in the nervous system and understand why presenilin mutations cause neurodegeneration. *C*. *elegans* provides a novel system for studying presenilin function because it does not produce Aβ peptides (Daigle & Li, 1993; McColl et al., 2012) and, thus, can help resolve the role of presenilin without Aβ accumulation confounding interpretations of presenilin function. Moreover, like the mammalian central nervous system, most *C*. *elegans* tissues are post‐mitotic. Therefore, they cannot use cell division to dilute damage organelles or protein aggregates and, hence, depend on efficient proteostatic pathways to clear faulty proteins and organelles. From our investigations, we have found that mutations in the *C*. *elegans* presenilin ortholog, SEL‐12, similar to mutations in human presenilin (Chan et al., 2000; Cheung et al., 2008; Oksanen et al., 2017; Stutzmann et al., 2004; Supnet & Bezprozvanny, 2011; Weidling & Swerdlow, 2020), results in altered ER calcium signaling, ER‐mitochondrial communication and mitochondrial function (Ashkavand et al., 2020; Sarasija & Norman, 2018). In *sel*‐*12* mutants, neuronal mitochondrial calcium levels are elevated, which results in mitochondrial hyperactivity and an increase reactive oxygen species (ROS) production. Importantly, reduction of ER calcium release or mitochondrial calcium uptake rescues neuronal as well as mitochondrial hyperactivation and prevents the neurodegenerative phenotypes observed in *sel*‐*12* mutants (Sarasija et al., 2018). Additionally, this altered ER‐mitochondrial calcium signaling and mitochondrial hyperactivity lead to profound defects in proteostasis, which has been found to be independent of gamma‐secretase activity (Ashkavand et al., 2020). Furthermore, examining cells from AD patients with PSEN1 mutations, others and we have found that mitochondrial hyperactivity promotes elevated ROS production that can be prevented by blocking mitochondrial calcium uptake (Oksanen et al., 2017; Sarasija et al., 2018). Thus, a vital question in understanding presenilin function concerns the identification of the molecular pathways connecting mitochondrial calcium uptake to neurodegeneration and loss of proteostasis.

Here, we report that the increased mitochondrial calcium uptake and associated mitochondrial activity accompanying SEL‐12 loss drives activation of the mTORC1 (mechanistic target of rapamycin complex I) pathway, which in turn impacts proteostasis and promotes neuronal dysfunction. We show that both genetic and pharmacological inhibition of mTORC1 greatly improves proteostasis in *sel*‐*12* mutants. Furthermore, we demonstrate that *sel*‐*12* mutants have reduced autophagosome formation that is dependent on mTORC1 signaling activity. Finally, we show that improvements to proteostasis, neuronal function, and neuronal health from mTORC1 inhibition in *sel*‐*12* mutants require the induction of autophagy. Our data indicate that elevated mitochondrial calcium uptake and mitochondrial activity resulting from SEL‐12 loss disrupts proteostasis through mTORC1 activation and mTORC1‐mediated suppression of autophagy, resulting in neurodegeneration.

## 2 RESULTS

### 2.1 *sel*‐*12* mutants show decreased autophagosome formation that is rescued by reducing mitochondrial calcium uptake

**FIGURE 1:**
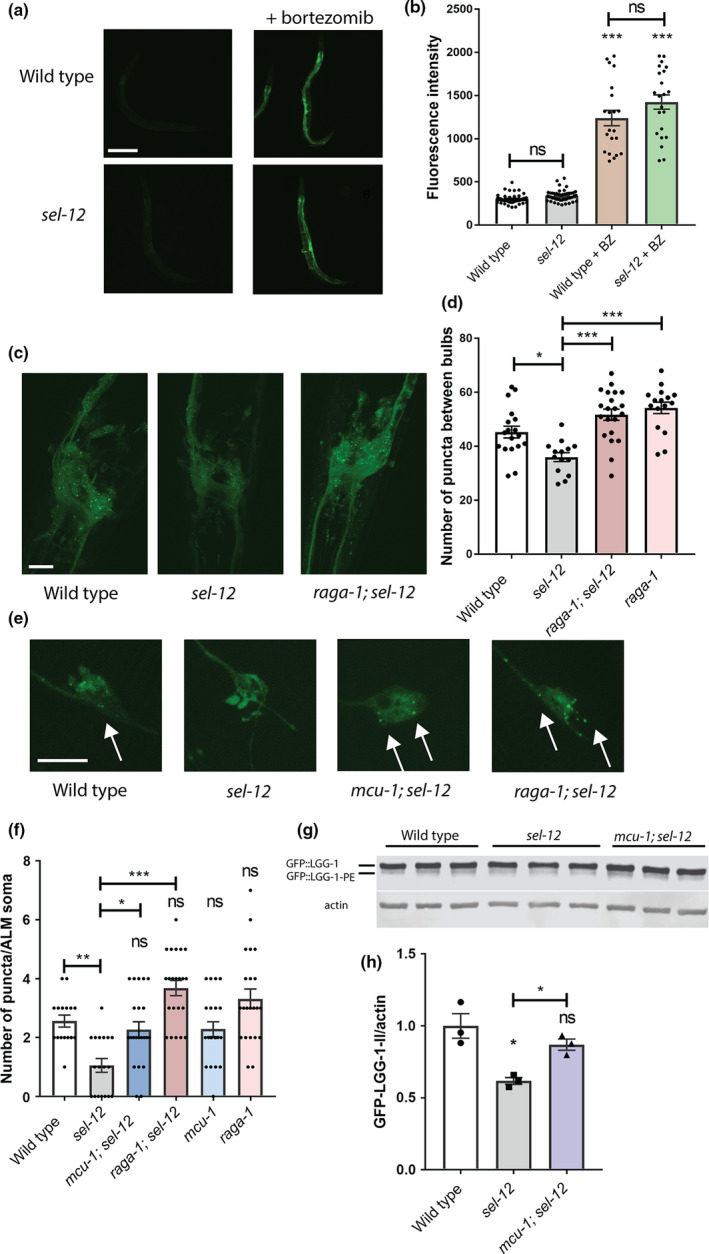
*sel*‐*12* mutants show reduced autophagosome formation that is restored by mTORC1 inhibition. (a and b) Representative images of wild‐type and *sel*‐*12 (ty11)* animals expressing *rpl*‐*28p*::ub(G76V)::GFP (*mgIs77*) treated with proteasome inhibitor (10 μM bortezomib) overnight (scale bar = 100 µm) (a) and quantification of GFP intensity (b). *n* is ≥25 animals. (c) Representative images of nerve ring neurons in animals expressing *rgef*‐*1p*::GFP::*lgg*‐*1* (*sqIs24*). Scale bar = 20 µm. (d) Quantification of GFP::LGG‐1 puncta in (c). (e) Representative images of ALM mechanosensory neurons in animals expressing *mec*‐*4p*::mCherry::GFP::*lgg*‐*1* (*rnyEx337*). Images show GFP positive puncta. Scale bar = 10 µm. (f) Quantification of GFP::LGG‐1 puncta (*rnyEx337*). Green puncta were counted within the soma as a measure of autophagosome number. (g and h) Western blot to determine the levels of PE‐conjugated (lipidated) GFP::LGG‐1 using an anti‐GFP antibody (g), and quantification of GFP::LGG‐1‐PE/actin (h). ns *p *> 0.05, *p < 0.05, ***p < .001 using one‐way ANOVA with Tukey's multiple comparison test. Error bars indicate mean ± SEM

Like other age‐related diseases, AD is associated with a general decline in proteostasis that contributes to the buildup of toxic protein aggregates, leading to neuronal dysfunction and death (Hartl et al., 2011; Morawe et al., 2012). It has been postulated that initial impairments to proteostasis pathways promote the accumulation and aggregation of misfolded proteins observed in neurodegenerative disorders, including the accumulation of the aggregation‐prone Aβ and tau, whose plaque deposition and tangle formation, respectively, are considered the hallmarks of AD (Balch et al., 2008; Chiti & Dobson, 2017; Hipp et al., 2014; Klaips et al., 2018; Kundra et al., 2017). Utilizing several models of proteotoxicity, we previously reported that *sel*‐*12* mutants have a severe defect in proteostasis resulting from elevated ER to mitochondrial calcium signaling (Ashkavand et al., 2020). Two major systems are responsible for degrading misfolded or damaged proteins and are critical for maintaining integrity of the proteome: the ubiquitin‐proteasome system and the autophagy‐lysosomal pathway. To investigate the activity of these pathways in *sel*‐*12* mutants, we first examined proteasome activity. As a reporter for proteasome function, we analyzed animals expressing ubiquitin (ub(G76V)) tagged to GFP, which is readily degraded by the proteasome in wild‐type animals, but accumulates if proteasome activity is perturbed (Lehrbach & Ruvkun, 2019) (Figure 1a). Indeed, unlike animals treated with a proteasome inhibitor (bortezomib), animals carrying a *sel*‐*12* null allele, *sel*‐*12(ty11)*, showed similarly low fluorescent intensity as wild‐type animals (Figure 1a,b), thus, indicating typical proteasomal degradation of the ub(G76V) tagged GFP. These data are consistent with normal proteasome activity of *sel*‐*12* mutants previously reported (Ashkavand et al., 2020).

Next to investigate the activity of autophagy‐lysosomal pathway in *sel*‐*12* mutants, we utilized GFP tagged LGG‐1 to visualize autophagosome formation. LGG‐1 is the *C*. *elegans* Atg8 ortholog, which is incorporated into and decorates pre‐autophagosomal and autophagosomal membranes and organizes into puncta during autophagy and, thus, is a widely used autophagy marker (Jia & Levine, 2007; Kang et al., 2007; Melendez et al., 2003). Compared to wild‐type animals, we observed a significant reduction in the number of GFP::LGG‐1 puncta in the hypodermal seam cells (Figure S1C–E) and body wall muscle (Figure S1F,G) of *sel*‐*12* mutants. To determine whether autophagosome formation is reduced within the nervous system of *sel*‐*12* mutants, we quantified puncta within the nerve ring neurons using animals expressing GFP::LGG‐1 driven under the pan‐neuronal *rgef*‐*1* promoter (Chang et al., 2017). We additionally quantified GFP::LGG‐1 puncta in the mechanosensory neurons by utilizing a strain expressing an mCherry‐GFP‐LGG‐1 reporter (Guha et al., 2020) within the mechanosensory neurons. Correspondingly, we found reduced GFP::LGG‐1 puncta in nerve ring neurons as well as in the mechanosensory neurons, suggesting an overall reduction in autophagosome formation within the neurons of *sel*‐*12* mutants (Figure 1c–f). To determine whether elevated ER‐mitochondrial calcium signaling in *sel*‐*12* mutants is responsible for decreased puncta formation, we introduced into the *sel*‐*12* mutant background a null mutation in the mitochondrial calcium uniporter, *mcu*‐*1*, which reduces mitochondrial calcium uptake (Sarasija et al., 2018; Xu & Chisholm, 2014) and has been shown to reduce mitochondrial calcium levels, neurodegenerative phenotypes, and proteostasis defects in *sel*‐*12* mutants (Ashkavand et al., 2020; Sarasija et al., 2018; Xu & Chisholm, 2014). We found that introduction of an *mcu*‐*1* null mutation in the *sel*‐*12* mutant background increases puncta formation to levels indistinguishable from wild‐type animals (Figure 1e,f, Figure S1E). This suggests that altered mitochondrial calcium signaling is responsible for reducing autophagosome formation in *sel*‐*12* mutants. As an alternative method to measure differences in levels of autophagosome formation, we immunoblotted for GFP in the GFP::LGG‐1 animals to assess lipidation of LGG‐1 with phosphatidylethanolamine (PE). When autophagy is induced, PE is conjugated to LGG‐1 to anchor it to the autophagosome. Thus, LGG‐1‐PE detection via Western blotting is a reliable method to measure autophagosomes (Springhorn & Hoppe, 2019). Consistent with this, GFP::LGG‐1‐PE levels significantly increase in *raga*‐*1* null mutants, which have been shown to have elevated autophagosome formation via inhibition of mTORC1 signaling (Robida‐Stubbs et al., 2012) (Figure S1A,B). *raga*‐*1* encodes the RagA GTPase ortholog, which is critical for mTORC1 activation. Consistent with our autophagosome puncta quantification, we found the amount of processed GFP::LGG‐1‐PE was significantly decreased in *sel*‐*12* mutants, and the level of processed GFP::LGG‐1‐PE was restored in *mcu*‐*1*; *sel*‐*12* animals (Figure 1g,h). Therefore, unlike proteasome activity in *sel*‐*12* mutants, these data suggest that autophagy is defective, which is consistent with previous observations of disrupted autophagy in *sel*‐*12* mutants as well as other models studying presenilin function (Ashkavand et al., 2020; Chong et al., 2018; Fedeli et al., 2019; Lee et al., 2010; Reddy et al., 2016) and also implicate a critical role of mitochondrial calcium in this defect.

### 2.2 Inhibition of mTORC1 in *sel*‐*12* mutants increases autophagosome formation

A central inhibitor of autophagy is the serine/threonine protein kinase mTORC1 signaling pathway (Kim & Guan, 2019; Liu & Sabatini, 2020). To investigate whether mTORC1 has a role in inhibiting autophagy in *sel*‐*12* mutants, we genetically ablated two key positive mediators of the mTORC1 pathway in *sel*‐*12* mutant animals. These include *raga*‐*1* and a gene encoding a key effector protein of mTORC1 signaling, ribosomal protein S6 kinase (*rsks*‐*1*). Analysis of GFP::LGG‐1 puncta in *raga*‐*1(ok386)*; *sel*‐*12* and *rsks*‐*1(ok1255)*; *sel*‐*12* double mutant animals reveals that *sel*‐*12* mutants with mTORC1 signaling inhibited, unlike *sel*‐*12* mutants alone, show robust accumulation of GFP::LGG‐1 puncta (Figure 1c–f, Figure S1C,D,F,G). Moreover, consistent with mTORC1 acting as a strong inhibitor of autophagy, blocking mTORC1 signaling resulted in elevated puncta formation and GFP::LGG‐1‐PE levels in wild‐type animals (Figure 1c–f, Figure S1A–D,F,G). Notably, a similar number of puncta is observed in *sel*‐*12* mutants with compromised mTORC1 signaling (Figure 1c–f, Figure S1C,D,F,G). These data indicate that *sel*‐*12* mutants have the capacity to carry out autophagy when mTORC1 signaling is disrupted. However, without mTORC1 inhibited, autophagosome formation is blunted in *sel*‐*12* mutants, suggesting a role of activated mTORC1 in mediating *sel*‐*12* phenotypes.

### 2.3 mTORC1 signaling is upregulated in *sel*‐*12* mutants

**FIGURE 2:**
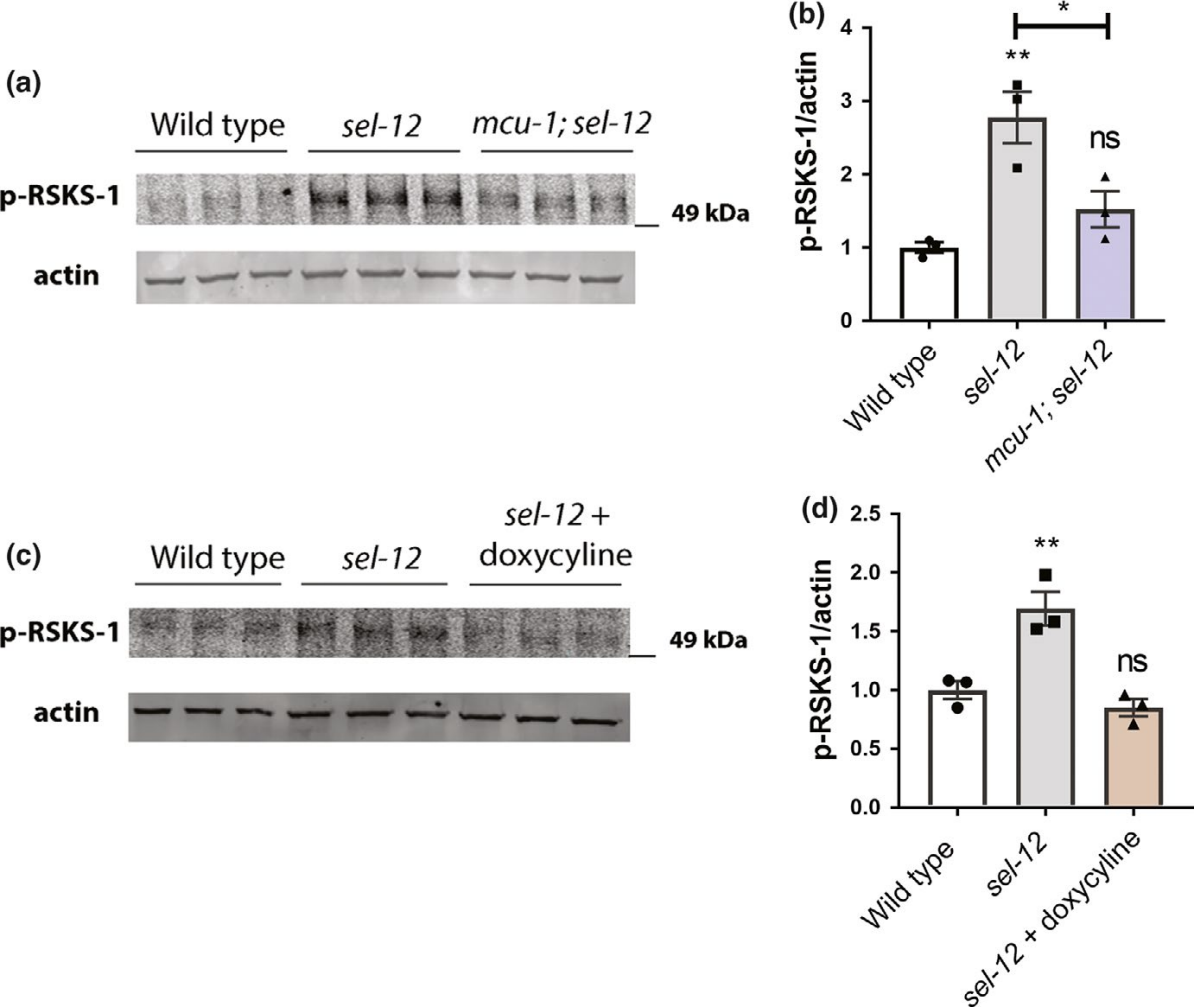
Reduction of mitochondrial calcium uptake or activity prevents mTORC1 hyperactivation in *sel*‐*12* mutants. (a and c) Western blot of p‐RSKS‐1 and actin (loading control), and (b and d) quantification of p‐RSKS‐1/actin. Error bars indicate ±SEM. ns *p *> 0.05, **p < 0.01 using one‐way ANOVA with Tukey's multiple comparison test. *n* ≥ 3

Given that the mTORC1 pathway is a central inhibitor of autophagy and is a critical metabolic sensor (Kim & Guan, 2019; Liu & Sabatini, 2020) and we have observed increased mitochondrial metabolic activity due to elevated ER to mitochondria calcium signaling in *sel*‐*12* mutants (Sarasija et al., 2018), we asked whether mTORC1 signaling is elevated in *sel*‐*12* mutants. To assess mTORC1 activity, we examined phosphorylation levels of the central mTORC1 target RSKS‐1/S6 kinase in *sel*‐*12* mutants. The p‐S6 kinase/p‐RSKS‐1 antibody was validated in *rsks*‐*1* null mutants (Figure S2A,B) and was previously shown to be a target of RAGA‐1 mediated mTORC1 signaling (Heintz et al., 2017). Strikingly, phosphorylated RSKS‐1 was significantly increased in *sel*‐*12* animals compared to wild‐type animals (Figure 2a,b), indicating that mTORC1 signaling is elevated in *sel*‐*12* mutants. Next, we sought to determine whether the increased mitochondrial activity observed in *sel*‐*12* mutants is leading to the elevation in mTORC1 signaling. Previously, we demonstrated that loss of *sel*‐*12* function promotes calcium uptake into the mitochondria from the ER, a process that increases mitochondrial activity and leads to the subsequent proteostatic collapse and neurodegeneration observed in *sel*‐*12* mutants (Ashkavand et al., 2020; Sarasija et al., 2018). Indeed, reducing ER calcium release or mitochondrial calcium uptake in *sel*‐*12* mutants, not only reduces oxidative phosphorylation, but suppresses the proteostasis defects and neurodegeneration phenotypes observed in *sel*‐*12* mutants (Ashkavand et al., 2020; Sarasija et al., 2018). To determine whether this altered calcium signaling pathway observed in *sel*‐*12* mutants is responsible for mTORC1 hyperactivation, we examined phospho‐RSKS‐1 levels in *mcu*‐*1*; *sel*‐*12* double mutants and found that introduction of the *mcu*‐*1* null mutation leads to reduced phopsho‐RSKS‐1 levels in *sel*‐*12* mutants compared to *sel*‐*12* mutants alone (Figure 2a,b). It is likely that mitochondrial calcium levels are responsible for changes to mTORC1 activation, as the *mcu*‐*1* mutation does not increase cytosolic calcium levels relative to the *sel*‐*12* mutation alone (Figure S3A,B). In addition to reducing mitochondrial calcium levels and consistent with calcium inducing mitochondrial respiration, the introduction of the *mcu*‐*1* null mutation into *sel*‐*12* mutants also decreases mitochondrial activity (Sarasija et al., 2018). This suggests that reducing mitochondrial activity decreases mTORC1 signaling in *sel*‐*12* mutants. To test this pharmacologically, we treated *sel*‐*12* worms with doxycycline, a mitochondrial protein translation inhibitor that reduces mitochondrial respiration (Moullan et al., 2015; Sarasija et al., 2018). Similar to blocking mitochondrial calcium uptake, *sel*‐*12* mutants treated with doxycycline abrogated the increase in phospho‐RSKS‐1 levels (Figure 2e,f). In addition, we treated *sel*‐*12* worms with 50 μM metformin, which is a mitochondria complex I inhibitor and reduces mitochondrial activity in *C*. *elegans* (Figure S2E,F) (Mor et al., 2020). Consistent with our other data, treatment of *sel*‐*12* mutants with metformin reduced p‐RSKS‐1 levels and mitochondrial activity (Figure S2C,D). Collectively, these data suggest that the altered ER‐mitochondrial calcium signaling in *sel*‐*12* mutants causes aberrant activation of the mTORC1 pathway by increasing mitochondrial activity.

### 2.4 Reduction of mTORC1 signaling suppresses neuronal defects in *sel*‐*12* mutants

**FIGURE 3:**
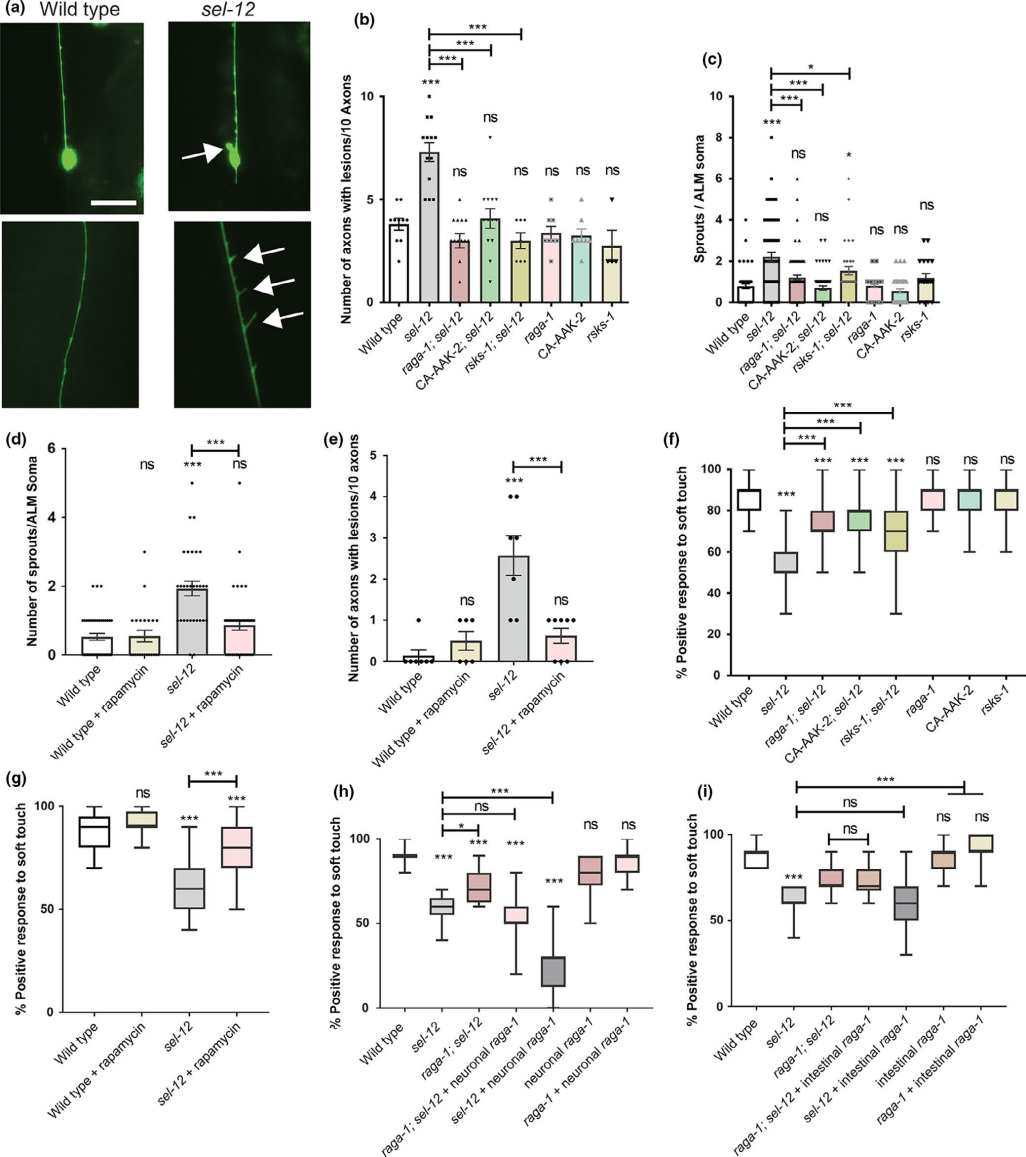
Inhibition of mTORC1 restores the structural integrity of mechanosensory neurons and improves mechanosensation in *sel*‐*12* mutants. (a) Representative images of wild‐type ALM mechanosensory neuron (top), *sel*‐*12* ALM with aberrant sprouts (top), wild‐type PLM axon (lower), and *sel*‐*12* PLM axon with aberrant lesions (lower) (scale bar = 10 µm). (b and c) Average number of aberrant ectopic sprouts per ALM soma (*n* ≥ 30 animals) (b) and number of aberrant lesions per 10 ALM/PLM axons (*n* ≥ 60 axons) (c) using the *bzIs166* [*mec*‐*4p*::mCherry] reporter. (d and e) Number of aberrant sprouts (d) and axonal lesions (e) in animals grown in presence or absence of rapamycin using the *zdIs5* [*mec*‐*4p*::GFP] reporter. (f and g) Soft touch assay quantifying percentage response to anterior and posterior soft touch. (*n* ≥ 50 animals.). (h) Soft touch assay in animals expressing *raga*‐*1* cDNA in neurons driven by the *rab*‐*3* promoter (*n* = 20 animals). (i) Soft touch assay in animals expressing *raga*‐*1* cDNA in the intestine driven by the *ges*‐*1* promoter (*n* = 20 animals). Error bars indicate mean ± SEM. ns *p *> 0.05, *p < .05, ***p < .001 using one‐way ANOVA with Tukey's multiple comparison test

We next asked whether mTORC1 activity contributes to the behavioral and neuronal defects seen with *sel*‐*12* loss. We first examined the structure of the *C*. *elegans* mechanosensory neurons, which show age‐dependent structural decline and neurodegeneration (Pan et al., 2011; Tank et al., 2011; Toth et al., 2012). Previously, we found that these structural aberrations associated with aging develop precociously in *sel*‐*12* mutants (Sarasija et al., 2018). Day 1 adult *sel*‐*12* mutants display numerous ectopic neurite sprouts stemming off the ALM neuronal soma which are absent in wild‐type animals, and also show defects in the structure of the ALM and PLM axons, exhibiting abnormal lesions at a higher frequency relative to wild‐type animals at day 1 (Figure 3a–c, Figure S4A–C). To determine whether mTORC1 inhibition can suppress these neuronal morphological defects, we examined mechanosensory neuron structure in *rsks*‐*1*; *sel*‐*12* and *raga*‐*1*; *sel*‐*12* animals, as well as in *aak*‐*2(uthIs248)*; *sel*‐*12* animals, which carry a mutation that results in constitutive activation of the catalytic subunit of 5′ adenosine monophosphate‐activated protein kinase (AMPK/AAK‐2), a global energy sensor and a major inhibitor of mTORC1 activity (Mair et al., 2011). *raga*‐*1*; *sel*‐*12*, *aak*‐*2(uthIs248)*; *sel*‐*12*, and *rsks*‐*1*; *sel*‐*12* animals each showed substantially improved neuronal morphology, with reduced ectopic neurite processes stemming off the soma (Figure 3c) and reduced frequency of lesions (Figure 3b), wave‐like processes (Figure S4B) and breaks (Figure S4C) in the ALM and PLM axons. In addition, we treated the *sel*‐*12* animals with rapamycin, a clinical grade drug that specifically inhibits mTORC1, and examined neuronal morphology. Consistent with the genetic manipulations, treatment with rapamycin showed similar improvements to the ALM soma (Figure 3d) and axonal structure (Figure 3e, Figure S4D,E).

To determine whether the structural improvements we observed in the mechanosensory neurons translate to functional improvement, we examined the effects of genetic and pharmacological mTORC1 inhibition on soft touch behavior, which is controlled by the mechanosensory neurons. Wild‐type day 1 adult animals, when touched on the anterior or posterior half of the body with an eyebrow hair, will reverse their progression and move away from the stimulus. Consistent with increased morphological defects in the mechanosensory neurons of aged animals, mechanosensation declines with age (Tank et al., 2011). This reduced response rate happens prematurely in *sel*‐*12* mutants and continues to worsen with age (Sarasija et al., 2018). Indeed, day 1 adult *sel*‐*12* mutants show pronounced defects in soft touch response (Figure 3f). Consistent with mechanosensory neuronal structural improvements, the *raga*‐*1*; *sel*‐*12*, *aak*‐*2(uthIs248)*; *sel*‐*12*, and *rsks*‐*1*; *sel*‐*12* double mutants all showed significant improvements to soft touch response (Figure 3f). Moreover, rapamycin treatment recapitulated these improvements (Figure 3g). Furthermore, this improvement is specific to mTORC1, as inhibition of mTORC2 signaling in *sel*‐*12* mutants showed no improvement to *sel*‐*12* mutant neuronal defects. Indeed, genetic ablation of RICTOR, *rict*‐*1*, which is required for the activation of mTORC2, in the *sel*‐*12* mutant background did not show significant improvements to mechanosensory neuron morphology or soft touch behavior (Figure S5A–E). It has been demonstrated that *hop*‐*1*, a second *C*. *elegans* presenilin orthologue, can compensate for egg‐laying defects caused by loss of *sel*‐*12* function (Eimer et al., 2002; Jarriault & Greenwald, 2002). Therefore, it is conceivable that improvements to mechanosensation in *sel*‐*12* mutants via these genetic manipulations are mediated through increased *hop*‐*1* expression. However, we found no difference in *hop*‐*1* mRNA expression in *raga*‐*1*; *sel*‐*12* and *mcu*‐*1*; *sel*‐*12* mutants compared to *sel*‐*12* alone, suggesting *hop*‐*1* upregulation does not facilitate the improvements in these animals (Figure S5F). Altogether, these data indicate that neurodegeneration in *sel*‐*12* mutants can be suppressed with mTORC1 inhibition.

Since we previously found that *sel*‐*12* acts cell autonomously in the nervous system to mediate soft touch response (Sarasija et al., 2018) and others have found a cell‐autonomous role of *sel*‐*12* in two interneurons that mediate thermotaxis (Wittenburg et al., 2000), we sought to investigate whether mTORC1 hyperactivity in the nervous system is promoting the nervous system defects observed in *sel*‐*12* mutants. We found that expression of *raga*‐*1* cDNA driven under the pan‐neuronal specific *rab*‐*3* promoter (Zhang et al., 2019) was sufficient to fully prevent any improvements to soft touch behavior in *raga*‐*1*; *sel*‐*12* animals (Figure 3h), indicating a cell‐autonomous role of mTORC1 hyperactivity in the nervous system of *sel*‐*12* mutants. Additionally, unlike in wild‐type animals, pan‐neuronal specific expression of *raga*‐*1* in the neurons of *sel*‐*12* animals significantly aggravated their touch defect. To determine whether mTORC1 also plays a non‐autonomous role in the neurodegeneration observed in *sel*‐*12* mutants, we similarly expressed *raga*‐*1* cDNA driven under the intestinal specific *ges*‐*1* promoter. However, we found that *raga*‐*1* expression in the intestine had no effect on soft touch behavior (Figure 3i). This suggests that mTORC1 activity primarily affects soft touch behavior in a cell‐autonomous manner. Overall, these data demonstrate a central role for neuronal mTORC1 activity in mediating and exacerbating the neurodegenerative behavioral defect in *sel*‐*12* mutants.

### 2.5 Inhibition of mTORC1 improves proteostasis in *sel*‐*12* mutants

**FIGURE 4:**
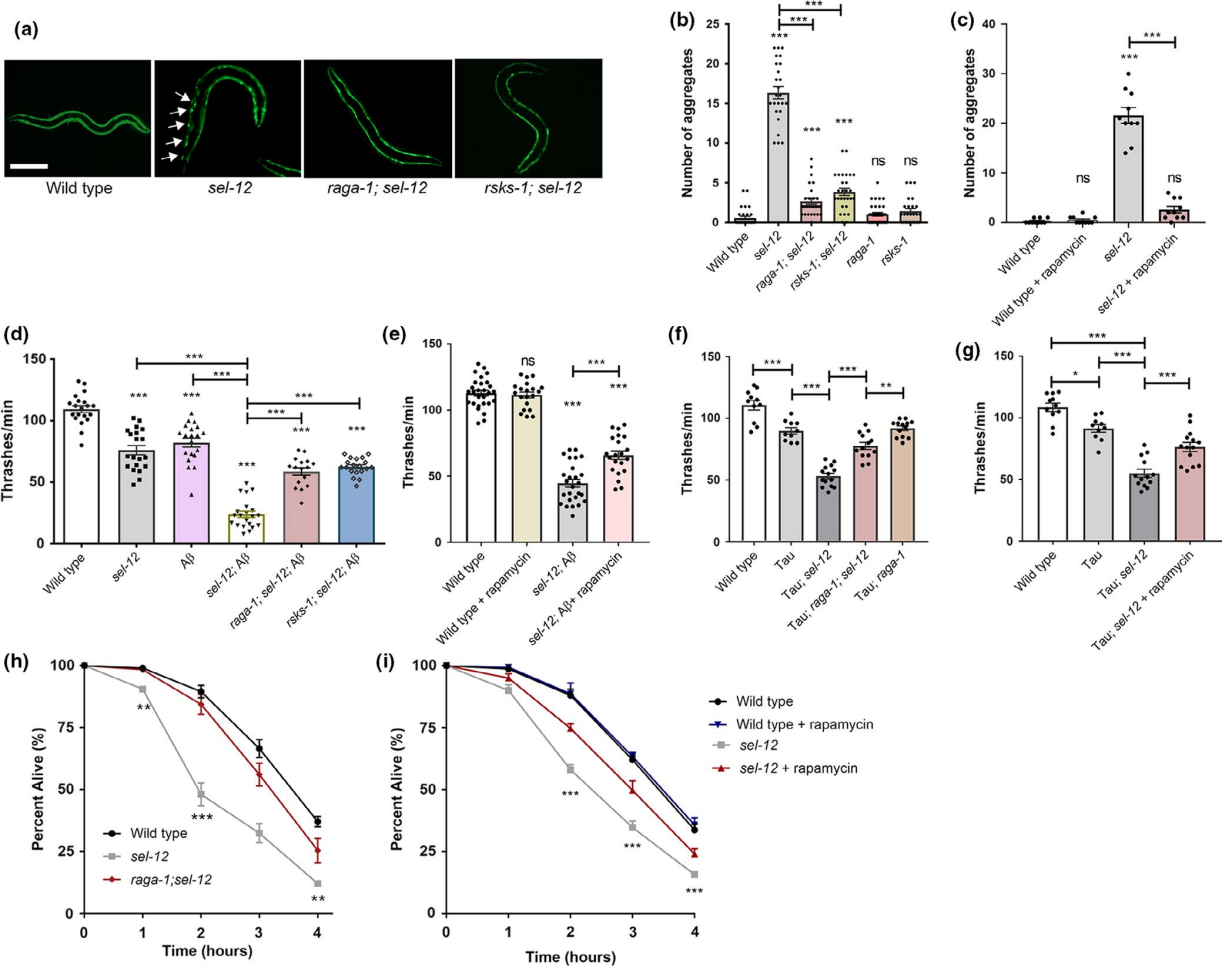
Inhibition of mTORC1 rescues loss of proteostasis in *sel*‐*12* mutants. (a) Representative images of animals expressing polyQ35::YFP (scale bar = 100 µm). (b and c) Mean number of polyQ aggregates observed in day 3 wild‐type, *sel*‐*12*, *raga*‐*1*; *sel*‐*12*, *rsks*‐*1*; *sel*‐*12*, *raga*‐*1*, and *rsks*‐*1* adults (b) or day 3 *sel*‐*12* adults following rapamycin treatment (c) (*n* = 30). (d and e) Mean thrashing rate in wild‐type, *sel*‐*12*, *raga*‐*1*; *sel*‐*12*, or *rsks*‐*1*; *sel*‐*12* mutants expressing human 1–42 Aβ *(dvIs100)* (d) or in 1–42 Aβ‐expressing *sel*‐*12* animals following rapamycin treatment (e) (*n* = 20.) (f and g) Mean thrashing rate in wild‐type, *sel*‐*12*, or *raga*‐*1*; *sel*‐*12* mutants expressing human V337M mutant tau (f) or in mutant tau‐expressing *sel*‐*12* animals following rapamycin treatment (g). (h and i) Sensitivity to heat stress. Animals were scored for survival after exposure to heat stress at 37℃. (50 animals per strain, repeated twice). Error bars indicate mean ± SEM. ns *p *> 0.05, *p < .05, **p < .01, ***p < .001 using one‐way ANOVA (a‐g) and two‐way ANOVA (h, i) with Tukey's multiple comparison test

mTORC1 hyperactivity may further explain impaired proteostasis in *sel*‐*12* animals. In fact, many studies show that modulation of mTORC1 activity widely impacts proteostasis (Su & Dai, 2017). To define a role of mTORC1 in the collapse of proteostasis in *sel*‐*12* mutants (Ashkavand et al., 2020), we first examined animals with body wall expression of polyglutamine (polyQ) Q35::YFP fusion protein *(rmIs132)*, which aggregates as the animals age (Morley et al., 2002). While expression of Q35::YFP remains soluble and evenly distributed in day 3 adult wild‐type animals, adult *sel*‐*12* mutants show premature Q35 aggregation by day 3. However, analyses of *raga*‐*1*; *sel*‐*12*, and *rsks*‐*1*; *sel*‐*12* animals show a significant reduction in polyQ aggregates at day 3 compared to *sel*‐*12* mutants, suggesting that mTORC1 inhibition improves proteostasis in these animals (Figure 4a,b). Consistent with these results, rapamycin treatment also reduced polyQ aggregates in day 3 adult *sel*‐*12* adult animals (Figure 4c).

As an alternate method to assess the state of proteostasis in *sel*‐*12* mutants, we examined animals expressing human Aβ1‐42 *(dvIs100)*, which generates proteostatic stress and causes progressive paralysis in the transgenic animals (McColl et al., 2012). Previously, we found that that *sel*‐*12* mutants expressing Aβ1‐42 have severely reduced motility relative to either mutant background alone, suggesting that the *sel*‐*12* mutation promotes Abeta1‐42 toxicity and enhances proteostasis defects (Ashkavand et al., 2020). To determine the effect of mTORC1 inhibition on motility in this background, we examined swimming behavior of *raga*‐*1*; *sel*‐*12*, and *rsks*‐*1*; *sel*‐*12* mutants expressing Aβ1‐42, and found they had significantly higher motility compared to *sel*‐*12* mutants expressing Aβ1‐42 (Figure 4d). Furthermore, treating *sel*‐*12* mutants expressing Aβ1‐42 with rapamycin showed similar improvements in motility (Figure 4e). To examine proteostasis specifically in the nervous system, we utilized animals that pan‐neuronally express human pathogenic V337M mutant tau, which impairs motility in the *sel*‐*12* mutant background (Figure 4f) (Ashkavand et al., 2020). We found inhibiting mTORC1 function through *raga*‐*1* mutation or rapamycin treatment improved locomotion in these transgenic animals (Figure 4f,g).

To evaluate the state of proteostasis of endogenous proteins, we subjected animals to heat stress (exposure to 37℃) to induce protein misfolding (Zevian & Yanowitz, 2014) and then examined animal survival. Previously, we found that *sel*‐*12* mutants have reduced resistance to heat stress and reduced survival (Ashkavand et al., 2020). Thus, we examined the survival rate of wild‐type, *sel*‐*12* and *raga*‐*1*; *sel*‐*12* mutants, as well as rapamycin, treated *sel*‐*12* animals after 1, 2, 3, and 4 h of exposure to 37℃. We found that the survival rate after heat stress at each time point was increased in the *sel*‐*12* mutants with mTORC1 signaling inhibited either genetically or pharmacologically relative to *sel*‐*12* mutants (Figure 4h,i). Altogether, these data suggest that mTORC1 impacts proteostasis in *sel*‐*12* mutants and that the defects in proteostasis due to loss of SEL‐12 are improved through mTORC1 inhibition.

### 2.6 Improvements to proteostasis and neuronal function through mTORC1 inhibition require the induction of autophagy

**FIGURE 5:**
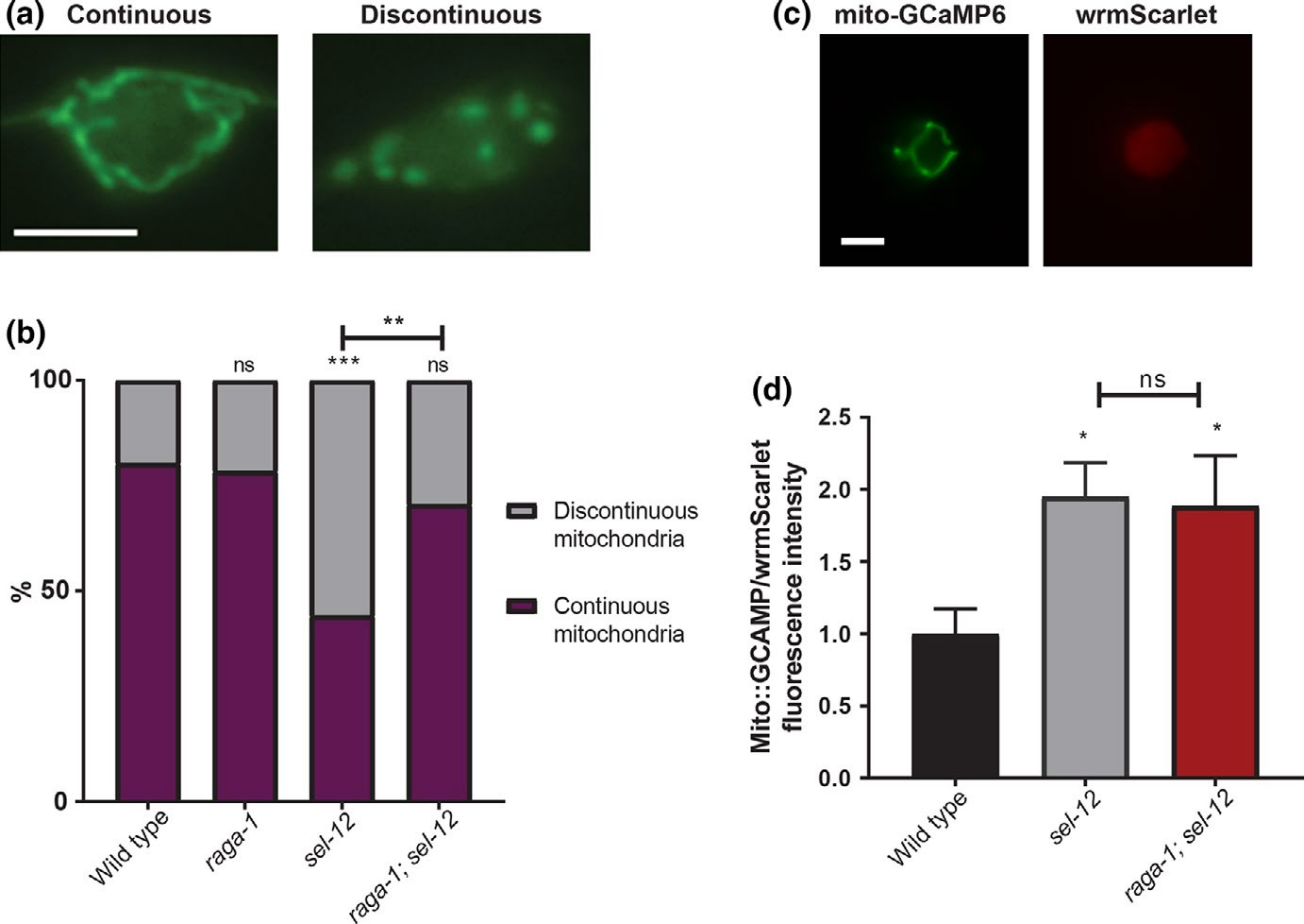
mTORC1 inhibition improves mitochondrial morphology but does not rescue elevated mitochondrial calcium in *sel*‐*12* mutants. (a) Representative images and quantification of ALM soma mitochondria in mechanosensory neurons using transgenic animals expressing mitochondrial localization signal tagged to GFP in the mechanosensory neurons (*jsIs609*) (scale bar = 5 µm). (*n* = 50). (b) Quantification of mitochondrial morphology (*n* ≥ 50 animals). (c and d) Representative images and quantification of mitochondrial matrix calcium levels using transgenic animals expressing mitochondrial‐targeted GCaMP6 and cytosolic wrmScarlet in the mechanosensory neurons to measure relative fluorescence intensity (scale bar = 5 µm). (*n* = 35). Error bars indicate mean ± SEM. ns *p *> 0.05, *p < .05, **p < .01***p < .001 using chi‐squared test (b) or one‐way ANOVA with Tukey's multiple comparison test (d)

To further investigate the mechanism by which mTORC1 contributes to proteostasis and neuronal defects in *sel*‐*12* mutants, we first examined the condition of mitochondrial morphology in *sel*‐*12* mutants with mTORC1 signaling abrogated. Previously, we found that *sel*‐*12* mutants have severe defects in mitochondrial morphology and function due to elevated mitochondrial calcium (Sarasija et al., 2018; Sarasija & Norman, 2015). To determine whether mTORC1 activity contributes to mitochondrial disorganization in *sel*‐*12* mutants, we examined *sel*‐*12* and *raga*‐*1*; *sel*‐*12* mutants expressing a mitochondrial localization signal (MLS) tagged with GFP in the mechanosensory neurons (Fatouros et al., 2012; Sarasija et al., 2018; Sure et al., 2018). We found that compared to *sel*‐*12* mutants, *raga*‐*1*; *sel*‐*12* double mutants had significantly improved mitochondrial organization, suggesting that elevated mTORC1 activity contributes to aberrant mitochondrial structure in *sel*‐*12* mutants (Figure 5a,b).

Next, since we previously found that disorganized mitochondrial structure in *sel*‐*12* mutants is caused by elevated ER to mitochondrial calcium signaling (Sarasija et al., 2018; Sarasija & Norman, 2015), we asked whether the improvements we observed in *raga*‐*1*; *sel*‐*12* mutants are due to reduced ER‐mitochondrial calcium signaling. Using a genetically encoded GCaMP6 calcium indicator localized to the mitochondrial matrix (Sarasija et al., 2018), we assessed mitochondrial calcium levels in the mechanosensory neurons. In accord with previous data, we found elevated calcium in the *sel*‐*12* mutants (Figure 5c,d). To investigate the possibility that the alteration to mitochondrial calcium is due to increased mitochondrial content, we used real‐time PCR to quantify mitochondrial DNA copy number versus nuclear DNA copy number. We also quantified the relative fluorescence intensity of animals expressing MLS::GFP. We found no difference in either mitochondrial DNA copy number or MLS::GFP fluorescence intensity between wild‐type and *sel*‐*12* mutants (Figure S6A,B), suggesting that elevated mitochondrial calcium is not due to increased mitochondrial content. Notably, we found that mitochondrial calcium levels were unchanged after mTORC1 inhibition (Figure 5c,d), indicating that mTORC1 activation is likely a downstream consequence of the altered calcium signaling observed in *sel*‐*12* mutants. These data are consistent with our p‐RSKS‐1 Western blot data showing that reduction of mitochondrial calcium uptake reduced p‐RSKS‐1 levels in the *sel*‐*12* mutant background (Figure 2a,b).

**FIGURE 6:**
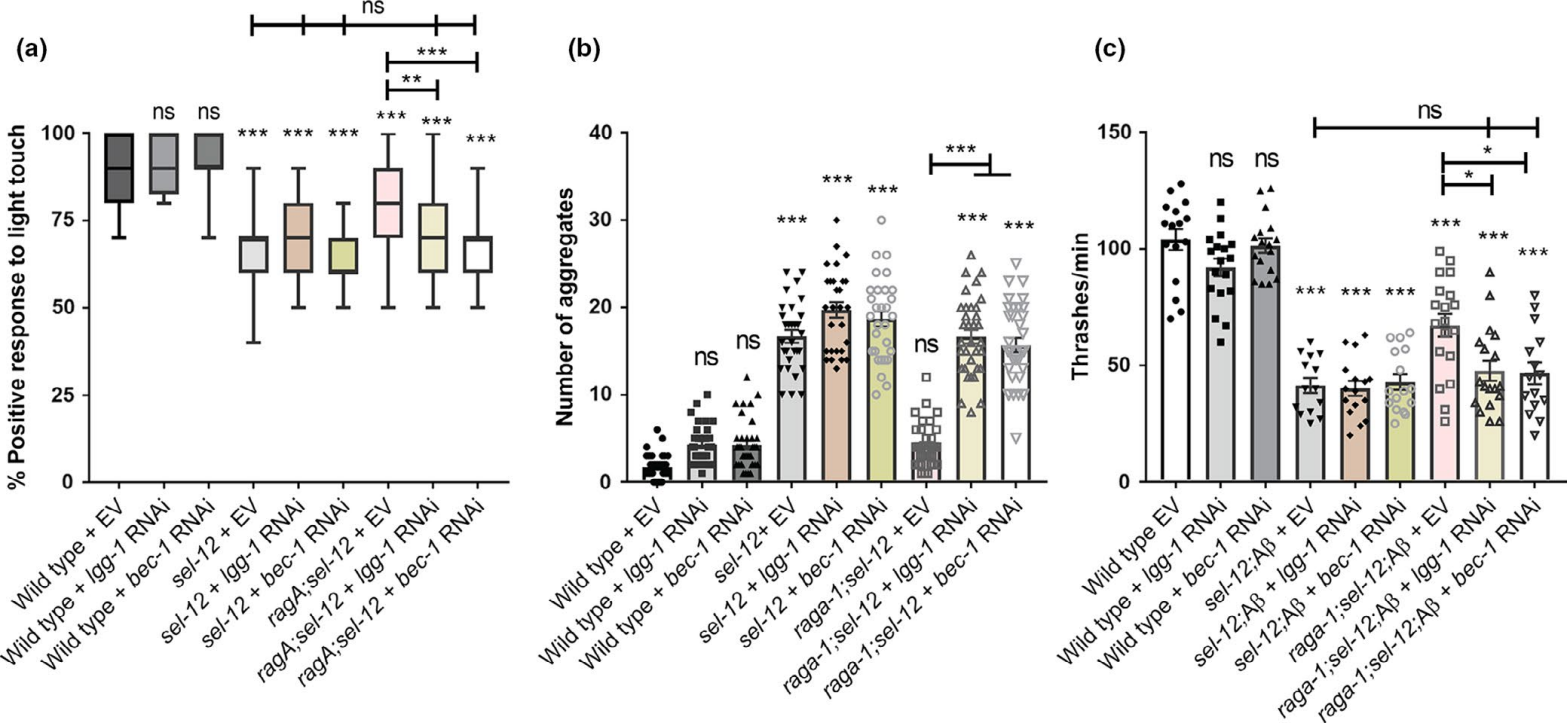
Rescue of proteostasis and behavior mediated by mTORC1 inhibition is dependent on autophagy. Percentage response to soft touch (a), number of thrashes per minute (b), or number of Q35 aggregates (c) in worms grown on EV, *lgg*‐*1*, or *bec*‐*1* RNAi. ns *p *> 0.05, *p < .05, **p < .01, ***p < .001 using one‐way ANOVA with Tukey's multiple comparison test. Error bars indicate mean ± SEM of ≥20 animals

Thus, since mTORC1 is likely not playing a role in mediating mitochondrial calcium signaling in the *sel*‐*12* mutants, it is possible mTORC1's inhibition of autophagy is responsible for the defects in proteostasis and neuronal function observed in *sel*‐*12* mutants. To determine whether the improvements in behavior and proteostasis through mTORC1 inhibition is primarily due to promoting autophagy, we knocked down inducers of autophagy *lgg*‐*1* and *bec*‐*1*, which encodes the *C*. *elegans* beclin 1 ortholog, using RNA interference (RNAi) (Hansen et al., 2008; Melendez et al., 2003). Importantly, RNAi directed to *lgg*‐*1* or *bec*‐*1* have been shown to impact autophagy in the mechanosensory neurons (Samara et al., 2008; Tóth et al., 2007). From these analyses, we found that the improvements to soft touch behavior in *raga*‐*1*; *sel*‐*12* mutants are abrogated when treated with *lgg*‐*1* or *bec*‐*1* RNAi (Figure 6a), and these animals resemble *sel*‐*12* mutant animals. Similarly, the improvements to proteostasis as measured by the number of Q35 aggregates in *raga*‐*1*; *sel*‐*12* double mutants are lost with either *lgg*‐*1* or *bec*‐*1* RNAi treatment (Figure 6b). Additionally, the increase in swimming rate is lost in *raga*‐*1*; *sel*‐*12* animals expressing Aβ1‐42 when treated with *lgg*‐*1* or *bec*‐*1* RNAi (Figure 6c). These data suggest mTORC1 primarily impacts proteostasis and neuronal function in *sel*‐*12* animals by inhibiting autophagy. Altogether, our data identify activation of mTORC1 as a critical pathway by which SEL‐12 loss results in neurodegeneration, and define an important role of the mTORC1 pathway in exacerbating the defects in proteostasis and autophagy following loss of SEL‐12.

## 3 DISCUSSION

AD is characterized by the pathophysiological buildup of protein aggregates such as amyloid plaques and neurofibrillary tangles. The decline in the ubiquitin‐proteasome and the autophagy‐lysosomal proteostasis pathways that are responsible for degrading misfolded or damaged proteins has been postulated to underlie age‐related diseases such as AD, Huntington's disease, Lewy body dementia, and Parkinson's disease (Kaushik & Cuervo, 2015; Labbadia & Morimoto, 2015; Yerbury et al., 2016). Here, we have shown that the premature decline in proteostasis in animals lacking SEL‐12/presenilin function is due, at least in part, to decreased autophagy. Our findings demonstrate that in *C*. *elegans* the loss of SEL‐12/presenilin function leads to elevated mTORC1 signaling and this increase is due to the disruption of mitochondrial calcium homeostasis and mitochondrial hyperactivity. Furthermore, this increase in mTORC1 signaling inhibits autophagy ultimately resulting in the collapse of proteostasis and progression of neuronal dysfunction. Thus, our data indicate a crucial function of SEL‐12/presenilin in mediating ER‐mitochondrial calcium homeostasis that is vital for proteostasis and disruption of this role leads to neurodegeneration in *C*. *elegans*.

Presenilins are a family of highly conserved proteins that are commonly found on endomembranes, such as the ER and endosomes (Smolarkiewicz et al., 2013). The functional role of the presenilin family on these endomembrane structures is not well understood. The best studied function of presenilin is its role as the catalytic subunit of the gamma secretase, an intramembrane aspartyl protease. In addition to presenilin, the gamma secretase is composed of three other well‐conserved components, including APH‐1, APH‐2/Nicastrin and PEN‐2 (Kimberly et al., 2003; De Strooper, 2003). Gamma‐secretase cleaves several type I transmembrane proteins most notably Notch and APP (Gertsik et al., 2015). It is presenilin's role in the proteolytic cleavage of APP that has drawn the most intensive research efforts. This is because mutations in *PSEN1* or *PSEN2* or mutations or duplications in *APP* cause early‐onset familial AD. Due to the accumulation of Aβ peptides in patients with AD and the ability of the gamma‐secretase to process APP to generate Aβ peptides, research on presenilin function has primarily focused on APP processing and the amyloid hypothesis, which centers on Aβ peptide accumulation causing AD. Despite intensive work on the amyloid hypothesis and a deep understanding into the processing of APP, the cause of AD is still not clear and a successful treatment for AD is not available. While smaller than the body of literature covering APP processing, there are many reports of gamma‐secretase independent functions of presenilin in regulating several biological pathways that are disrupted in AD, such as calcium homeostasis, mitochondrial and lysosomal function, and autophagy (Area‐Gomez et al., 2012; Bezprozvanny & Mattson, 2008; Cheung et al., 2008; Lee et al., 2010; Neely et al., 2011; Nelson et al., 2007; Reddy et al., 2016).

Presenilins found on the ER are enriched in membrane fractions that are associated with mitochondrial contact, the mitochondrial‐associated membranes (Area‐Gomez et al., 2009). Interestingly, disruption of presenilin function results in more frequent ER‐mitochondrial contacts and signaling shared between these organelles, which leads to altered lipid synthesis and calcium uptake into the mitochondria (Area‐Gomez & Schon, 2013; Rossi et al., 2019). This altered calcium uptake results in mitochondrial functional changes and elevated oxidative stress (Oksanen et al., 2017; Sarasija et al., 2018; Sarasija & Norman, 2015). In *C*. *elegans*, this oxidative stress leads to neuronal dysfunction, which can be suppressed by limiting ER calcium release, mitochondrial calcium uptake, or mitochondrial activity (Sarasija et al., 2018). Importantly, the ER‐mitochondrial activity of presenilin has been shown to be gamma‐secretase independent (Area‐Gomez et al., 2012; Ashkavand et al., 2020; Sarasija et al., 2018). Moreover, in *C*. *elegans*, we have found that this altered ER‐mitochondrial calcium signaling promotes the collapse of proteostasis in *sel*‐*12* mutants, which can be reversed by reducing ER calcium release or mitochondrial calcium uptake (Ashkavand et al., 2020).

Here, we find that the proteostasis defects associated with loss of SEL‐12/presenilin are due to a reduction of autophagy caused by elevated mTORC1 activity. Both *in vivo* models of AD and post‐mortem brain samples from AD patients have revealed hyperactivated mTORC1 signaling (Bhaskar et al., 2009; Caccamo et al., 2011; Li et al., 2005; Tramutola et al., 2016). mTORC1 activity promotes cellular growth and metabolism by activating many anabolic pathways, such as the biosynthesis of protein and lipids, and inhibiting catabolic processes, such as autophagy. Despite reports indicating mTORC1 hyperactivation in AD, the cause of mTORC1 activation is not known. Several studies have suggested Aβ peptides directly or indirectly activate mTORC1 (Caccamo et al., 2011; Ma et al., 2010), and other studies have suggested that the activation of mTORC1 is an enhancer of Aβ generation and deposition (An et al., 2003; Cai et al., 2012). Interestingly, several laboratories have shown that hyperactive mTORC1 contributes to tau pathology (e.g. neurofibrillary tangles), which in addition to amyloid plaques, is a classic hallmark of AD (An et al., 2003; Caccamo et al., 2013; Khurana et al., 2006). In *C. elegans sel*‐*12* mutants, we find that altered ER‐mitochondrial calcium signaling and the likely concomitant mitochondrial hyperactivity increases mTORC1 signaling, which subsequently leads to decreased autophagy. This in turn promotes proteostatic stress in *sel*‐*12* mutants driving neuronal dysfunction. Since *C*. *elegans* does not generate Aβ peptides (Alexander et al., 2014), the mechanism leading to mTORC1 activation in driving neurodegeneration in *C*. *elegans* is not mediated through Aβ peptides but through altered mitochondrial calcium homeostasis and mitochondrial hyperactivity. Indeed, we demonstrate that reduction of mitochondrial calcium or mitochondrial activity in *sel*‐*12* mutants reduces mTORC1 signaling. Importantly, we have shown that reduction of ER calcium release or mitochondrial calcium uptake rescues the proteostasis defects and neurodegeneration observed in *sel*‐*1*2 mutants (Ashkavand et al., 2020; Sarasija et al., 2018). Nevertheless, it is unclear how calcium and mitochondrial hyperactivity are promoting mTORC1 activity.

Calcium uptake into the mitochondrial matrix is critical for calcium buffering and influencing mitochondrial metabolic activity (e.g., generation of NADH, ATP, and superoxide). Alterations in mitochondrial calcium uptake can lead to oxidative stress or calcium overload triggering cell death (Finkel et al., 2015; Starkov et al., 2004; Williams et al., 2013). Interestingly, several recent studies have revealed a role of increased mitochondrial calcium levels in promoting neurodegenerative phenotypes (Ham et al., 2020; Jadiya et al., 2019; Ludtmann et al., 2019; Müller et al., 2018; Verma et al., 2017). For example, in Drosophila and zebrafish, it was found that mutations in the genes encoding the PINK1 ortholog results in elevated mitochondrial calcium, and when mitochondrial calcium was reduced in these mutants neurodegeneration was prevented (Lee et al., 2018; Soman et al., 2019). Moreover, it was recently demonstrated in a mouse model of AD that neuronal mitochondrial have increased calcium levels that precedes neurodegeneration and that inhibition of mitochondrial calcium influx prevents neurodegeneration (Calvo‐Rodriguez et al., 2020). However, the role calcium, mitochondrial function, and/or mTORC1 have in these observations is unclear. Of note, it has been previously shown that reduction of mitochondrial calcium increases AMP:ATP ratio and activates AMPK, which stimulates autophagy, however, unlike our observations, through an mTORC1 independent mechanism (Cardenas et al., 2010). Nonetheless, we have shown that constitutive activation of AMPK, similar to *mcu*‐*1* and *raga*‐*1* mutants, can suppress the nervous system defects found in *sel*‐*12* mutants, suggesting that reduction of mitochondrial calcium uptake may stimulate AMPK activity and, thus, inhibit mTORC1 signaling in *sel*‐*12* mutants.

A limitation of our study is that we did not directly examine mTORC1 or mitochondrial activity in the nervous system. Nevertheless, we found expression of neuronal *raga*‐*1* cDNA prevented improvements to soft touch behavior in *raga*‐*1*; *sel*‐*12* (Figure 3h) mutants, unlike expression of intestinal *raga*‐*1* (Figure 3i), suggesting that neuronal mTORC1 activity is perturbed in *sel*‐*12* mutants. There is also evidence that dysregulated mitochondrial activity occurs at a cell‐autonomous level in presenilin mutant cells. Data from FAD PSEN1 mutant fibroblasts showed that inhibiting mitochondrial calcium uptake alleviated the elevated oxidative respiration and ROS present within these cells (Sarasija et al., 2018). Similarly, PSEN1 mutant iPSC‐derived astrocytes were shown to exhibit higher respiration resulting in elevated ROS (Oksanen et al., 2017). Nonetheless, whether neuronal mTORC1 and mitochondrial activity are elevated in *sel*‐*12* mutants remains a standout question. In this study, in addition to analyzing the *mcu*‐*1* knockout, which reduces mitochondrial calcium content and mitochondrial respiration (Sarasija et al., 2018; Xu & Chisholm, 2014), we used doxycycline and metformin to reduce mitochondrial activity and found that all three of these manipulations resulted in improved neuronal function in *sel*‐*12* mutants, suggesting mitochondrial hyperactivity is the cause of *sel*‐*12* mutant neuronal dysfunction. Yet, it should be noted that inhibition of OCR by metformin has also been shown to reduce mTORC1 activation through alternative mechanisms (Chen et al., 2017; Wu et al., 2016). Thus, further investigation is necessary to define the link between mitochondrial hyperactivity and mTORC1 activation in the nervous system of *sel*‐*12* mutants.

Our data show that loss of SEL‐12/presenilin function causes proteostatic collapse and neurodegeneration, at least in part, by the hyperactivation of mTORC1 and the ensuing inhibition of autophagy in *C*. *elegans*. Of note, hyperactivation of mTORC1 signaling is associated with several neurological disorders, such as autism, AD, and tuberous sclerosis, and suppression of mTORC1 using compounds that inhibit mTOR signaling has been shown to be an effective treatment in several clinical trials (Caccamo et al., 2014; Crino, 2016; Kaur & Sharma, 2017). Using *C*. *elegans*, we have found that treatment with rapamycin, a classic mTORC1 inhibitor, improves the proteostasis and neurodegeneration defects associated with *sel*‐*12* mutants. Additionally, several studies have demonstrated the effectiveness of rapamycin on ameliorating neuropathology in mouse AD models overexpressing amyloid beta or hyperphosphorylated Tau (Lin et al., 2013; Ozcelik et al., 2013; Siman et al., 2015; Spilman et al., 2010). Thus, these data suggest that treatment with mTOR inhibitors may provide some therapeutic benefit to patients suffering from AD. In summary, our study shows that loss of SEL‐12/presenilin results in mTORC1 activation, caused by exacerbated mitochondrial calcium uptake and concomitant mitochondrial hyperactivity, which further contributes to loss of proteostasis and neurodegeneration in *sel*‐*12* mutants.

## 4 MATERIALS AND METHODS

### 4.1 *C. elegans* maintenance and strains

For all experiments, *C*. *elegans* strains were grown on *E*. *coli* OP50 seeded NGM plates at 20℃. Animals were age synchronized by bleaching gravid worms to obtain the eggs, which were then incubated in M9 for 24–48 h before being allowed to hatch. Afterward, L1 larvae were grown to adulthood on NGM plates for further experiments. Day 1 adults were analyzed for all experiments unless otherwise indicated. For Q35 aggregation experiments, L4 animals were sterilized by moving them to plates containing 0.5 mg/ml 5‐fluorouraci1‐2′‐deoxyribose (Sigma) until the age required for the experiment was reached.

The following strains were used: N2 was the wild type, *egl*‐*19(n2368)* IV, *raga*‐*1(ok386)* II, *rict*‐*1(ft7)* II, *rsks*‐*1(ok1255)* III, *sel*‐*12(ty11)* X, *spr*‐*4(by105)* I, *bzIs166* [*mec*‐*4p*::mCherry], *zdIs5* [*mec*‐*4p*::GFP + *lin*‐*15*(+)] I, *dvIs100* [*unc*‐*54p*::A‐beta‐1–42::*unc*‐*54* 3′‐UTR + *mtl*‐*2p*::GFP], *goeIs22* [*mec*‐*4p*::SL1::GCaMP3.35::SL2::mKate2::*unc*‐*54* 3′UTR + *unc*‐119(+)], *jsIs609* [*mec*‐*4p*::MLS::GFP], *mgIs77* [*rpl*‐*28p*::ub(G76V)::GFP + *unc*‐*119*(+) + *myo*‐*2p*::mCherry] V, *rmIs132* [*unc*‐*54p*::Q35::YFP], *sqIs13* [*lgg*‐*1p*::GFP::*lgg*‐*1* + *odr*‐*1p*::RFP], *sqIs24* [*rgef*‐*1p*::GFP::*lgg*‐*1* + *unc*‐*122p*::RFP], *rnyEx337* [pSKG7 (*mec*‐*4p*:: mCherry::gfp::*lgg*‐*1*, pCFJ90(*myo*‐*2p*::mCherry), pCI(pha‐1+)], *uthIs248* [*aak*‐*2p*::*aak*‐*2*(genomic aa1‐321)::GFP::*unc*‐*54* 3′UTR + *myo*‐*2p*::tdTOMATO], *takEx612* [*mec*‐*7p*::mito‐GCaMP6f::SL2::wrmScarlet], *takEx677* [*ges*‐*1p*::3X FLAG::*raga*‐*1* cDNA::gpd‐2 SL2::mCherry::*unc*‐*54* 3′UTR], *wbmEx238* [*rab*‐*3p*::*raga*‐*1* cDNA::SL2::mCherry::*unc*‐*54* 3′UTR]. Genotypes were determined by PCR and DNA sequencing.

### 4.2 Analysis of neuronal morphology

The structure of the mechanosensory neurons was observed in either *mec*‐*7p*::*GFP(zdIs5*) or *mec*‐*4p*::*mCherry(bzIs166*)‐expressing animals. Presence or absence of wave‐like bending in the axon, lesions sprouting off the axon, or a beading‐like pattern indicating breaks was determined. The number of sprouts stemming from the ALM soma was counted. The worms were immobilized in 0.3% sodium azide on 2% agarose pads and imaged using the 63× oil objective on a Zeiss Axio Observer microscope equipped with an Andor Clara charged‐coupled device (CCD) camera, and Metamorph software was used to compile the images.

### 4.3 Mitochondrial organization analysis

The organization of the mitochondria in the ALM mechanosensory neuronal soma was observed in animals expressing *mec*‐*4p*::MLS::GFP(*jsIs609*). Animals were immobilized in 1 M levamisole on 2% agarose pads, then imaged using 100× oil objective on a Zeiss Axio Observer microscope equipped with an Andor Clara CCD camera, and Metamorph software was used to compile the images. Mitochondrial organization was scored on a binary scale, where continuous mitochondria did not show breaks and discontinuous mitochondria appeared fragmented and showed breaks.

### 4.4 Heat stress assay

For each strain, approximately 50 age‐synchronized day 1 adults were placed in a water bath at 37℃ for 1, 2, 3, and 4 h, then allowed to recover at 20℃ for 2 h. Animals were scored as dead if they did not show any movement when prodded with a wire pick.

### 4.5 Mechanosensation assay

Day 1 adults' response to soft touch was determined using an eyebrow hair attached to a Pasteur pipette, with ten touches per worm, and with each stroke alternating across the anterior and posterior half of the worm as previously described (Sarasija et al., 2018). A positive response was scored when the animal moved away from the hair. The mean percentage of positive responses per worm was then determined.

### 4.6 Locomotion assay

Day 1 adults were placed in a well that was coated with 2% agarose and filled with 150 μL M9 buffer. A single worm was transferred to an unseeded plate to remove OP50, then picked into the M9 buffer. After waiting 1 min for the worm to acclimate, the number of full body bends was counted for 1 min.

### 4.7 Quantitation of polyQ aggregates

*unc*‐*54p*::Q35::YFP(*rmIs132*)‐expressing day 3 adult animals were immobilized in 0.1 µm diameter polystyrene microspheres (Polysciences) on 2% agarose pads and were imaged using the 10× objective on a Zeiss Axio Observer microscope equipped with an Andor Clara CCD camera. Metamorph software was used to compile the images. The number of polyQ aggregates was counted, each aggregate determined as a structure fully discernible from any surrounding aggregates.

### 4.8 Autophagosome assays

We utilized animals expressing the *lgg*‐*1p*::GFP::*lgg*‐*1* (*sqIs13*) construct to label autophagosomes (Lapierre et al., 2013). Animals were immobilized in 0.1 µm diameter polystyrene microspheres (Polysciences) on 2% agarose pads. The number of aggregates per visible seam cell or per visible muscle cell was counted. Animals were viewed at 63× magnification using Zeiss AxioObserver microscope captured with an Andor Clara CCD camera, and Metamorph software was used to compile the images. All imaging of autophagosomes in the nervous system was captured at 60× magnification using a Nikon laser‐scanning confocal microscope with NIS Elements software. *rgef*‐*1p*::GFP::*lgg*‐*1* (*sqIs24*) animals were used to image autophagosomes in the nerve ring, while imaging of autophagosomes in the mechanosensory neurons was conducted in animals expressing a mCherry‐GFP‐Atg8/LGG‐1 reporter in the mechanosensory neurons (Guha et al., 2020). Z‐stack images were acquired at 0.25 µm slice intervals for all animals. The total number of puncta between the two pharyngeal bulbs was counted within a 15 µm slice to measure total autophagosome number in the nerve ring.

### 4.9 Proteosome function assay

*rpl*‐*28p*::ub(G76V)::GFP(*mgIs77*)‐expressing day 1 adult animals were immobilized in 1 M levamisole on 2% agarose pads and were imaged using the 10× objective on a Zeiss Axio Observer microscope equipped with an Andor Clara charged‐coupled device CCD camera. Metamorph software was used to compile the images. The fluorescence intensity was quantified using ImageJ.

### 4.10 Western blot analysis

Day 1 adult worms were washed with PBS, then lysed via sonication in RIPA buffer with protease (Roche) and phosphatase inhibitors (Roche). For p‐RSKS‐1 analysis, half the sample was lysed in RIPA buffer with protease inhibitors to determine protein concentration using a BCA assay (Pierce), while the other half was lysed in 2× Laemmli sample (BioRad) buffer containing 5% beta‐mercaptoethanol for use in the assay. 15 μg of each sample was separated with a 10% tris‐glycine gel (BioRad), or with 12% tris‐glycine gel (BioRad) for GFP::LGG‐1 samples, then transferred to a 0.2 μm nitrocellulose membrane (Invitrogen). The membrane was incubated in TBS with primary antibody overnight (phospho‐Drosophila p70 S6 Kinase (Thr398), 1:500, Cell Signaling #9209; GFP, 1:1000, Cell Signaling #2555; and beta‐actin, 1:1000, MP Biomedicals #8691002). Fluorescent tagged secondary antibodies were used (IRDye 800CW Goat anti‐rabbit (LI‐COR), 1:20,000 and IRDye 680RD Goat anti‐mouse, 1:20,000 (LI‐COR)). The blot was imaged using LiCor Odyssey CLx infrared imaging system and quantified with the Odyssey Image Studio software.

### 4.11 Calcium imaging

Mitochondrial calcium was measured in the mechanosensory neurons in animals expressing *mec*‐*7p*::mito‐GCaMP6f::SL2::wrmScarlet as previously described (Sarasija et al., 2018). In brief, animals were immobilized on 1 M levamisole on 2% agarose pads. Images were taken using a 100× objective lens on a Zeiss Axio Observer microscope equipped with an Andor Clara CCD camera, and images were compiled with Metamorph software. Fluorescence intensity of GCaMP6 was normalized to wrmScarlet fluorescent intensity, which was used as an expression control, and quantified using ImageJ. Cytoplasmic calcium levels were measured similarly in the mechanosensory neurons of animals expressing *mec*‐*4p*::SL1::GCaMP3.35::SL2::mKate2, with fluorescence intensity of GCaMP3.35 normalized to mKate2 intensity using ImageJ.

### 4.12 RNAi

RNAi was delivered by feeding as previously described (Timmons & Fire, 1998). L1 animals were grown to adulthood on NGM plates seeded with HT115 bacteria expressing *lgg*‐*1* or *bec*‐*1* double‐stranded RNA, both from the Ahringer library (Kamath et al., 2003), or empty RNAi feeding vector. RNAi bacteria strains were verified by PCR and DNA sequencing. Furthermore, to show specificity *lgg*‐*1(RNAi)* and *bec*‐*1(RNAi)* treatment abolished or reduced GFP::*lgg*‐*1*, respectively.

### 4.13 Drug treatments

Rapamycin was prepared in dimethyl sulfoxide (DMSO) and added to plate agar at 100 µM. Equivalent DMSO was added to control plates. Bortezomib (LC Laboratories) was prepared in DMSO and added to plate agar at 10 μM. Doxycycline was added as described (Sarasija et al., 2018), with doxycycline prepared in and added to plates at a concentration of 10 µg/ml. Equivalent DMSO was also used in control plates. For rapamycin, doxycycline, and bortezomib treatments, L4 animals were grown on treated plates overnight, and the assays performed the following day on day 1 adults. 50 μM of metformin or equivalent volume of DMSO was added onto plate agar with OP50, similar to described (Mor et al., 2020) and OP50 was heat‐killed (65℃ for 30 min) prior to seeding. L1 animals were grown on metformin or control plates until day 1 of adulthood.

### 4.14 Oxygen consumption rate

The Seahorse XFp Extracellular Flux Analyzer was used to determine oxygen consumption rate (OCR) as described (Houtkooper et al., 2013; Sarasija & Norman, 2019). Each assay compared the OCR between two treatment groups, with each group measured in triplicate. Five measurements were each taken for basal OCR and maximal OCR per treatment group. 25 μM FCCP was used to induce maximal OCR, and 40 mM sodium azide was used to block mitochondrial respiration to distinguish between non‐mitochondrial and mitochondrial OCR.

### 4.15 Quantitative PCR

Total RNA was extracted from N2, *sel*‐*12 (ty11) X*, *spr*‐*4(by105)* I, *raga*‐*1 (ok386)* II, *raga*; *sel*‐*12 (ty11)*, *mcu*‐*1 (tm6026)* IV, and *mcu*‐*1*; *sel*‐*12 (ty11)* using RNeasy mini kit (Qiagen, Germany) according manufacturer's manual. After reverse transcription, the quantitative PCR was performed in triplicate with SYBR Green master mix (Applied Biosystems, USA) and StepOnePlos Real‐time PCR system (Applied Biosystems) with the following PCR conditions: 10 min at 95℃ followed by 40 cycles of 95℃ for 15 s and 60℃ for 1 min. Primers used for PCR are as follows:

**Table:** 

β‐Actin	(F) gctggacgtgatcttactgattacc (R) gtagcagagcttctccttgatgtc
*hop‐1* (Lu et al., 2014)	(F) tgctggtttactcagttttc (R) aaatgaaatcgctgttaatg
*ama‐1*	(F) tggaactctggagtcacacc (R) catcctccttcattgaacgg
*ctb‐1*	(F) ctaggttatattgccacggtg (R) caataaacatctctgcatcacc

The level of *hop*‐*1* mRNA was normalized by the level of actin as a housekeeping gene. The fold change in mRNA was calculated by using 2−ΔΔCt.

The relative levels of nuclear vs. mitochondrial DNA were determined as previously described (Gonzalez‐Hunt, 2016). Worms were lysed with REDExtract‐N‐Amp Tissue PCR Kit (Sigma Aldrich) to obtain DNA. Primers for *ama*‐*1* were used as a measurement of nuclear DNA copy number and *ctb*‐*1* for mitochondrial DNA copy number. nucDNA and mtDNA content were also calculated using 2−ΔΔCt.

### 4.16 Statistical analyses

All statistical analyses were conducted using Graph Pad Prism software. A *p* value of less than 0.05 is considered to be significant. Statistical difference was determined using a Student's *t* test for comparing two variables and a one‐way analysis of variance with a Tukey post hoc analysis for comparing more than two variables.

## CONFLICT OF INTEREST

The authors declare no competing interests.

## AUTHOR CONTRIBUTIONS

KCR and KRN conceived the project and designed experiments. KCR, ZA, SS, and JTL performed experiments. RS provided technical support. KRN and KCR analyzed and interpreted the data and wrote the manuscript.

## Supporting information

Fig S1

Fig S2

Fig S3

Fig S4

Fig S5

Fig S6

Fig S1‐S6

## Data Availability

The datasets used and/or analyzed during the current study are available from the corresponding author on reasonable request.
