# Supplementary figures and images for: Increased mitochondrial calcium uptake and concomitant mitochondrial activity by presenilin loss promotes mTORC1 signaling to drive neurodegeneration
Source: Aging Cell. 2021 Sep 9;20(10):e13472. doi: 10.1111/acel.13472 (PMC8520713; doi:10.1111/acel.13472)

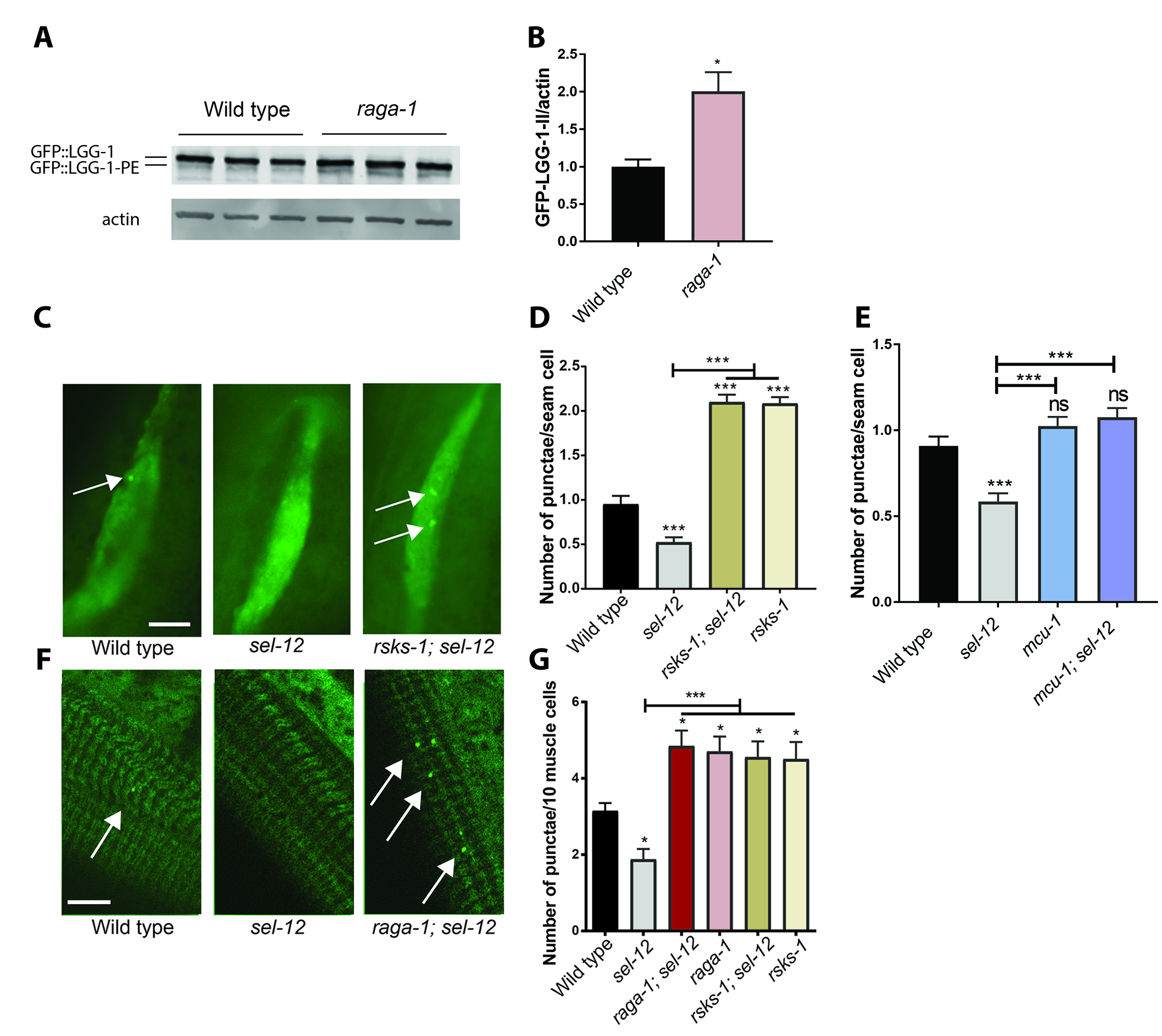

Supplement: Supplementary file 1 — Fig S1 [file ACEL-20-e13472-s001.tif]

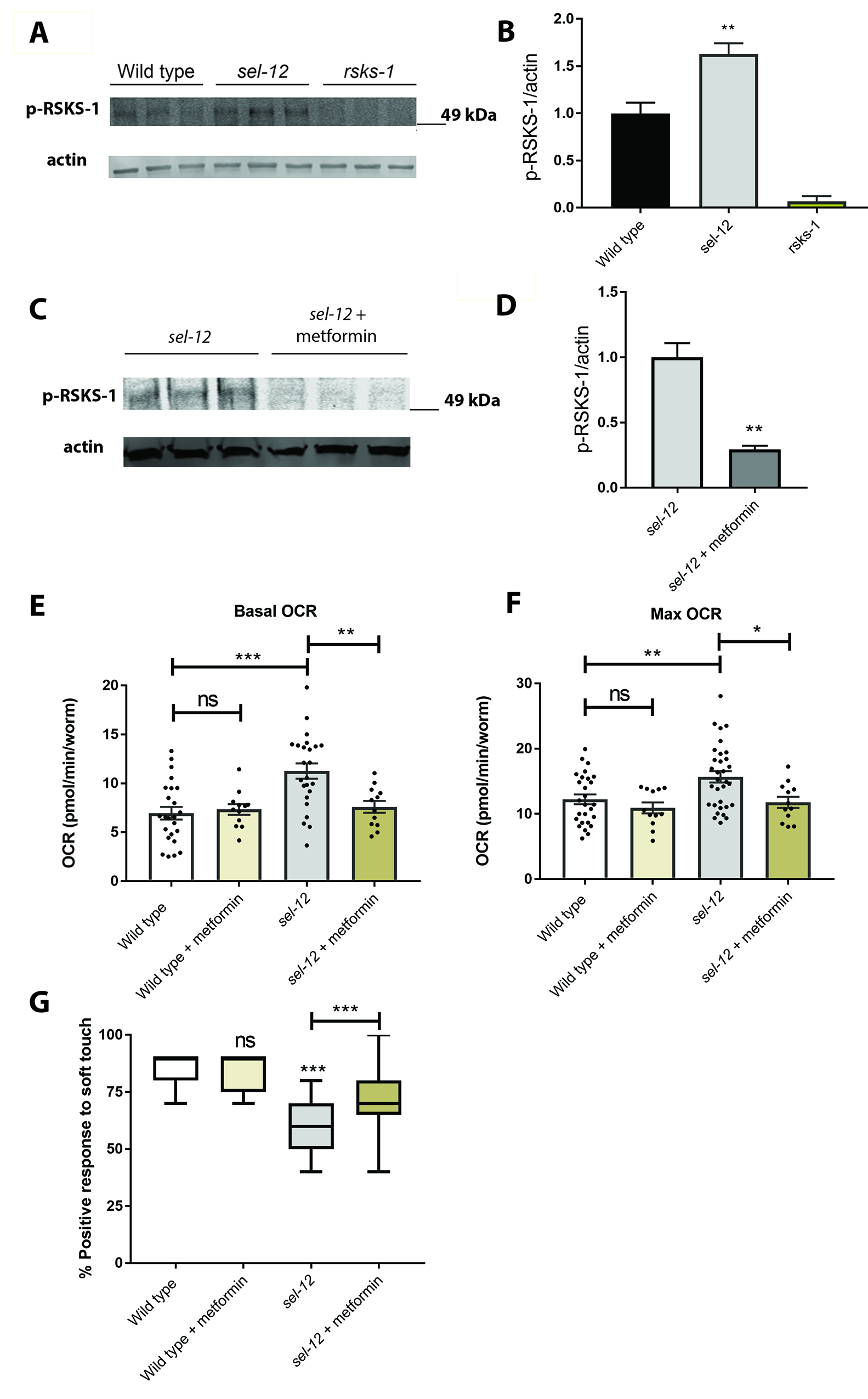

Supplement: Supplementary file 2 — Fig S2 [file ACEL-20-e13472-s002.tif]

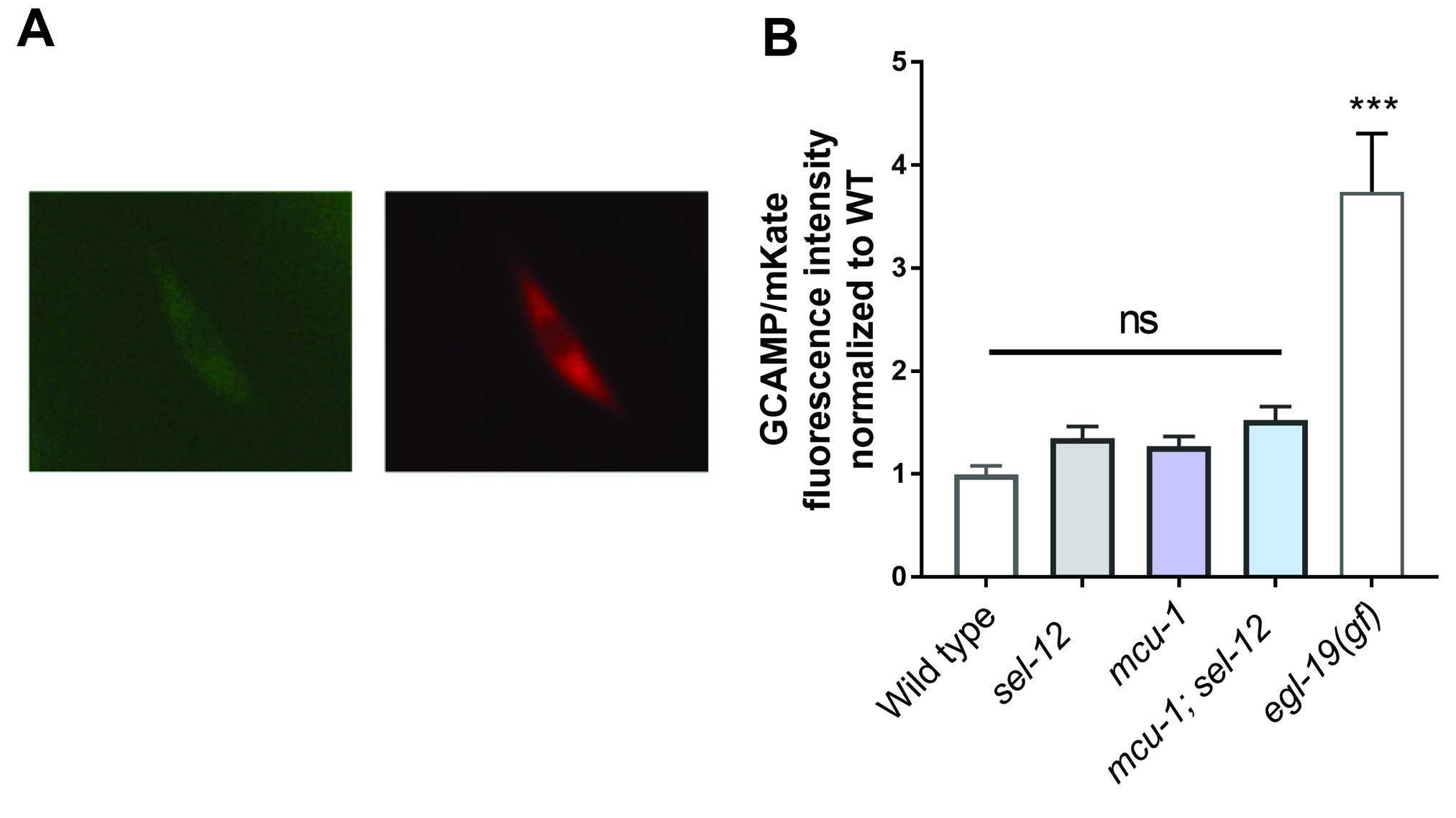

Supplement: Supplementary file 3 — Fig S3 [file ACEL-20-e13472-s007.tif]

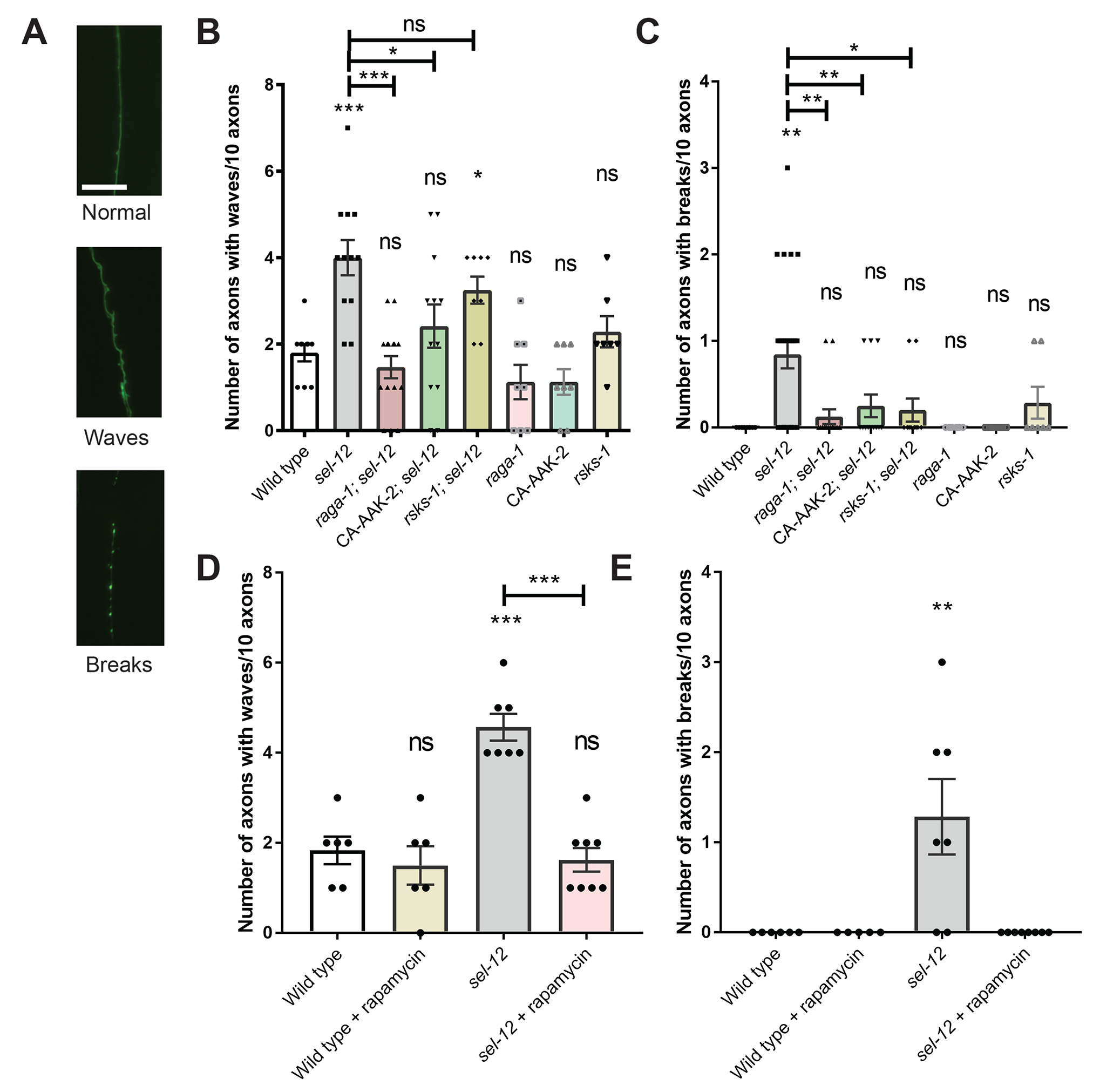

Supplement: Supplementary file 4 — Fig S4 [file ACEL-20-e13472-s003.tif]

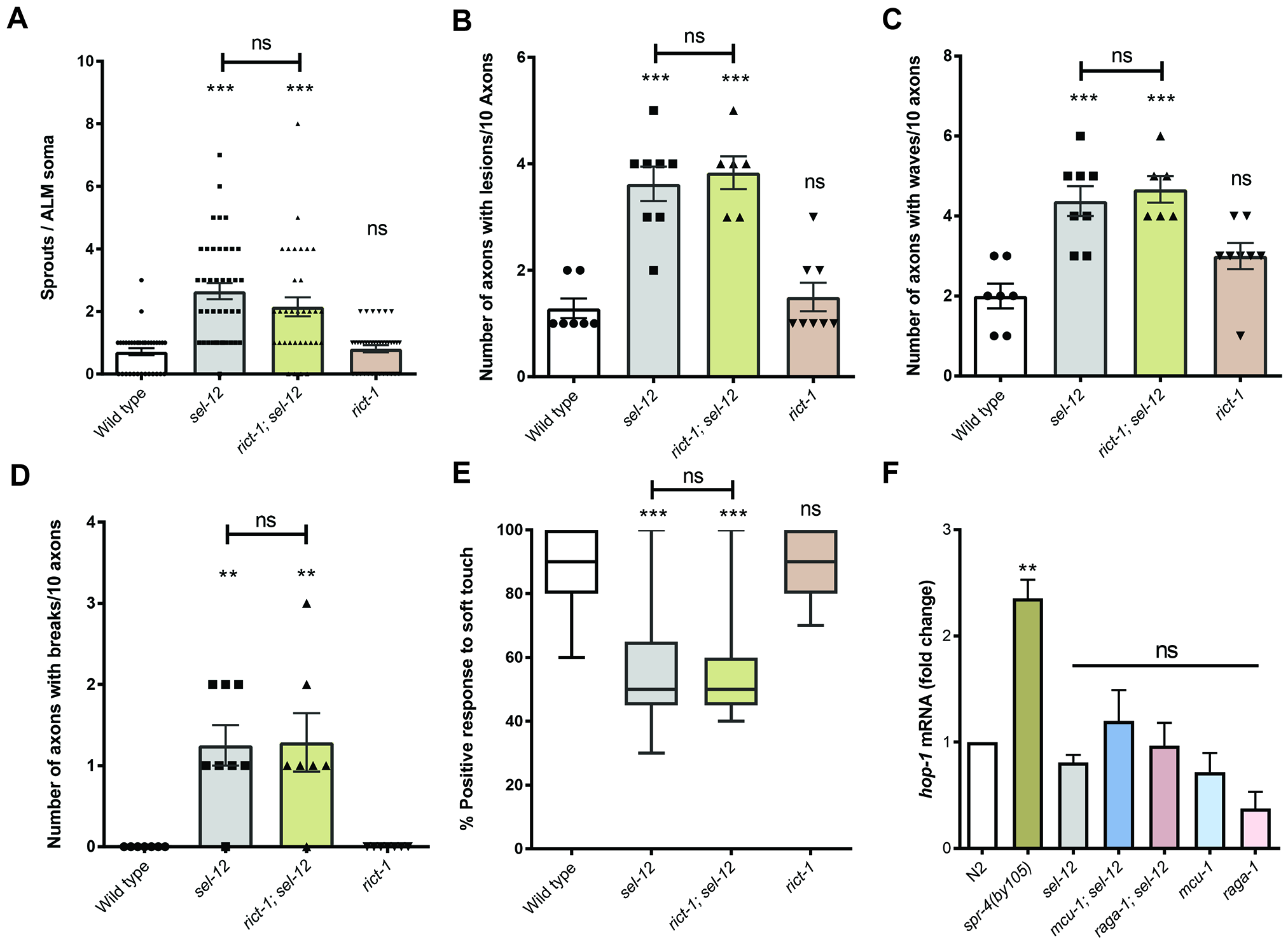

Supplement: Supplementary file 5 — Fig S5 [file ACEL-20-e13472-s006.tif]

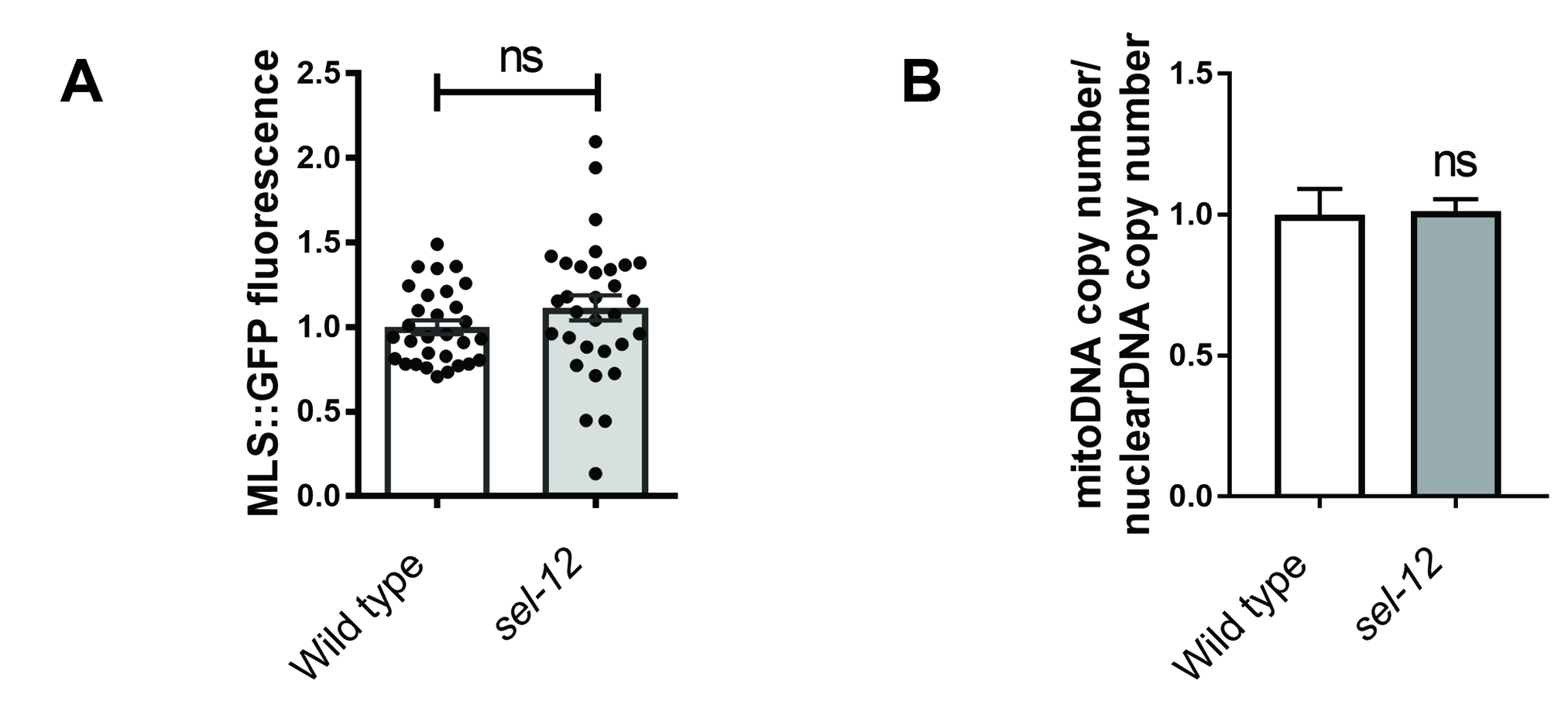

Supplement: Supplementary file 6 — Fig S6 [file ACEL-20-e13472-s004.tif]
