# Supplementary material for: Increased mitochondrial calcium uptake and concomitant mitochondrial activity by presenilin loss promotes mTORC1 signaling to drive neurodegeneration
Source: Aging Cell. 2021 Sep 9;20(10):e13472. doi: 10.1111/acel.13472 (PMC8520713; doi:10.1111/acel.13472)
Supplement: Supplementary file 7 — Fig S1‐S6 [file ACEL-20-e13472-s005.docx]

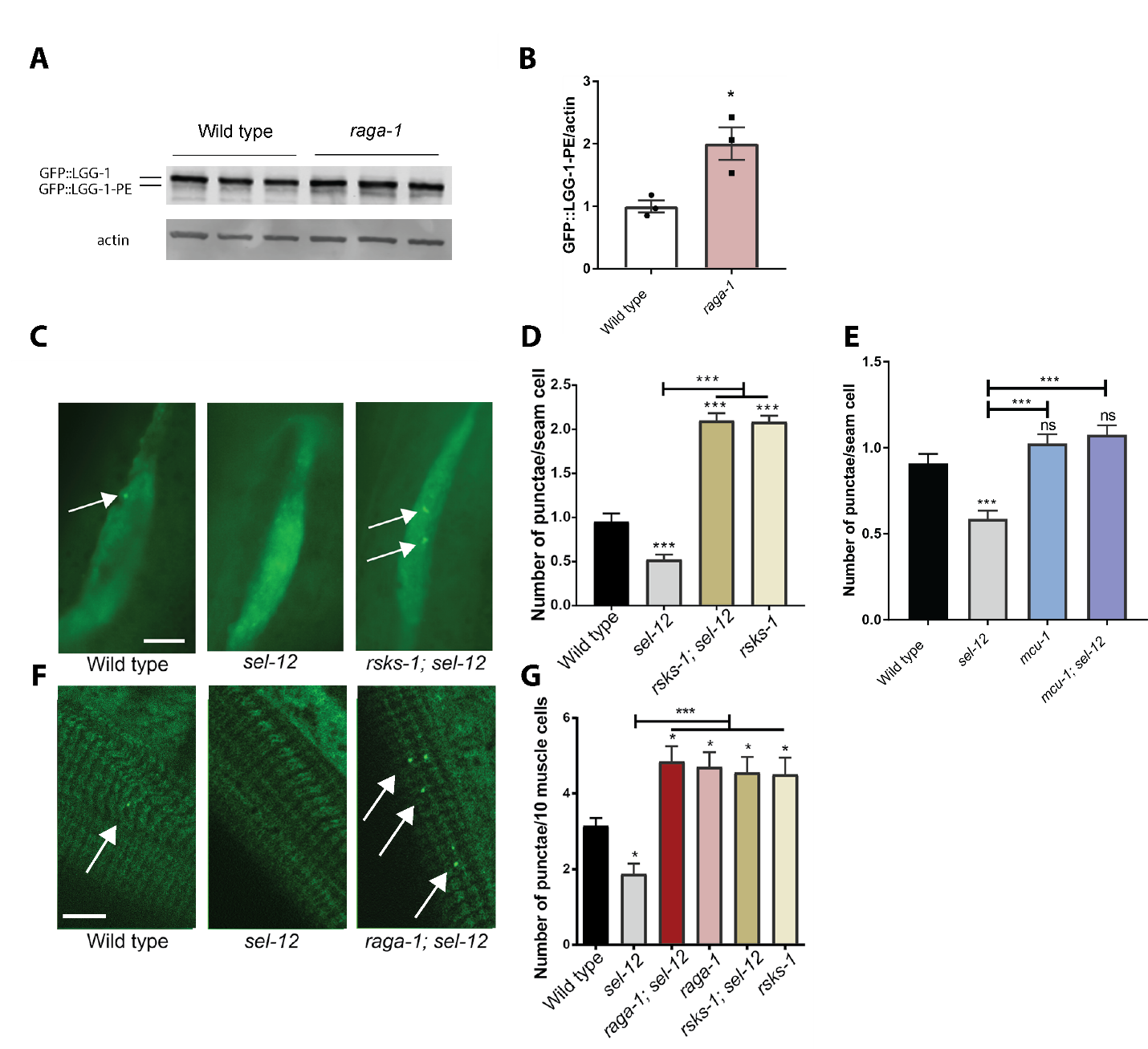


Figure S1


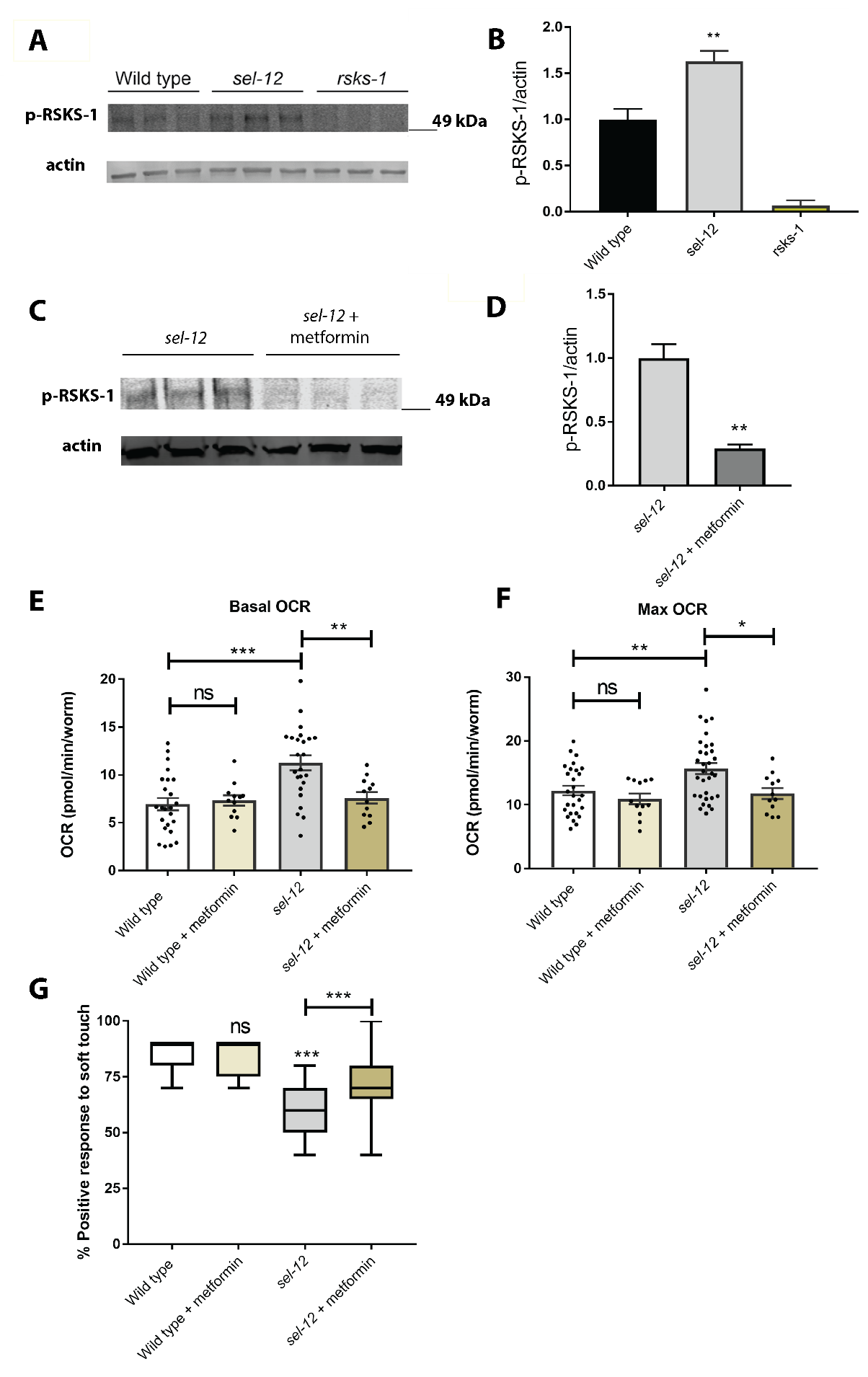


Figure S2


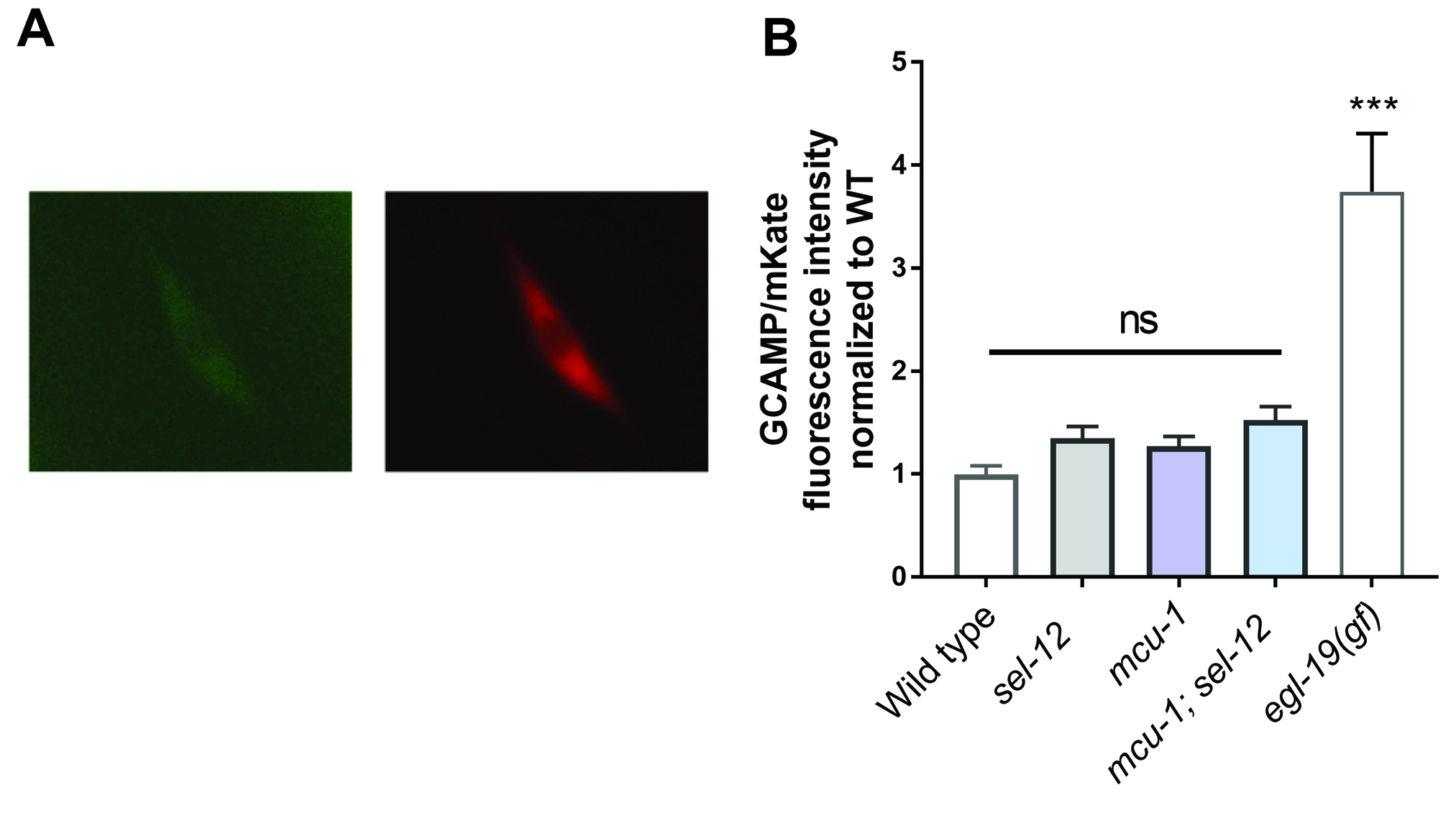


Figure S3


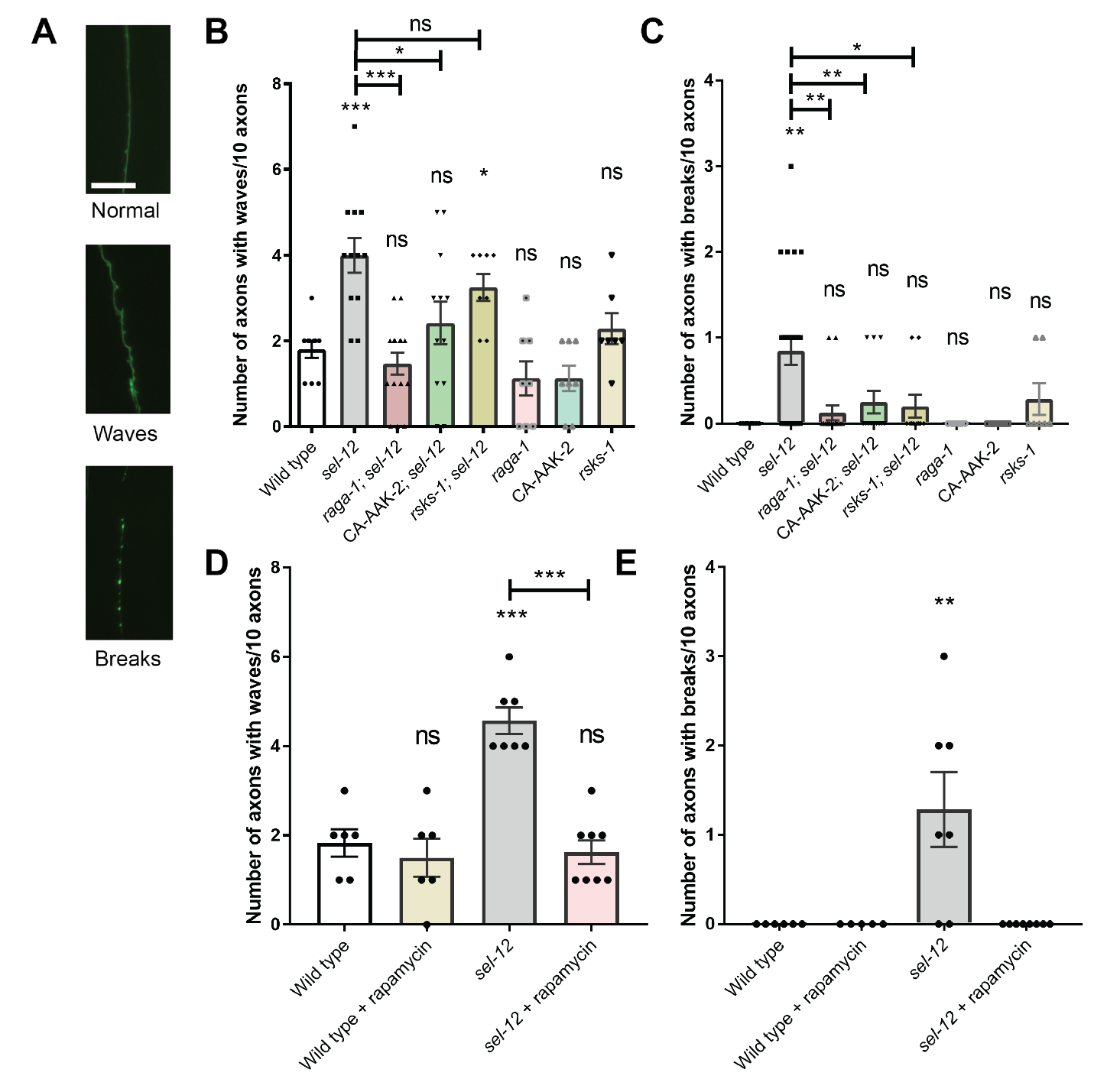


Figure S4


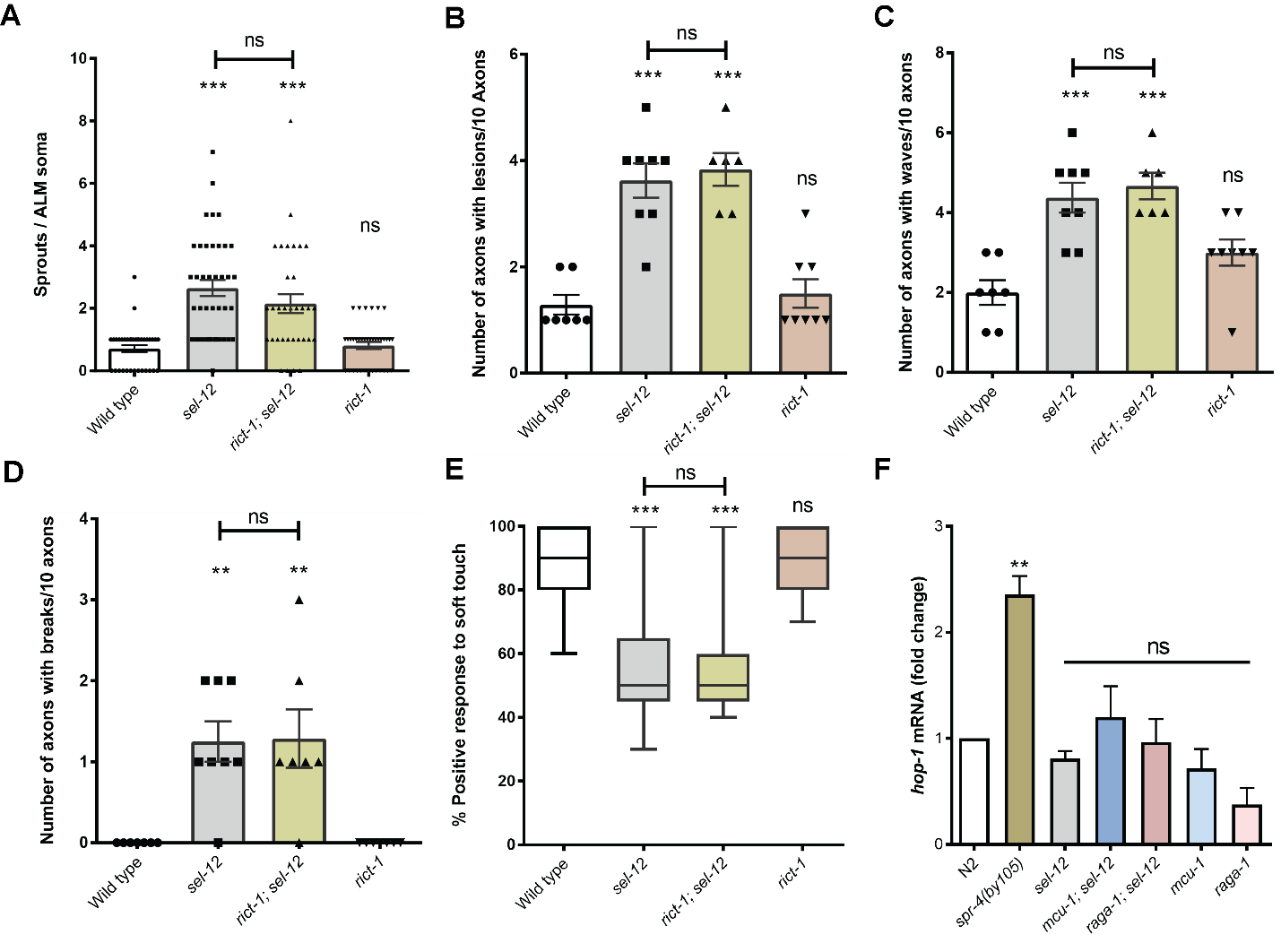


Figure S5


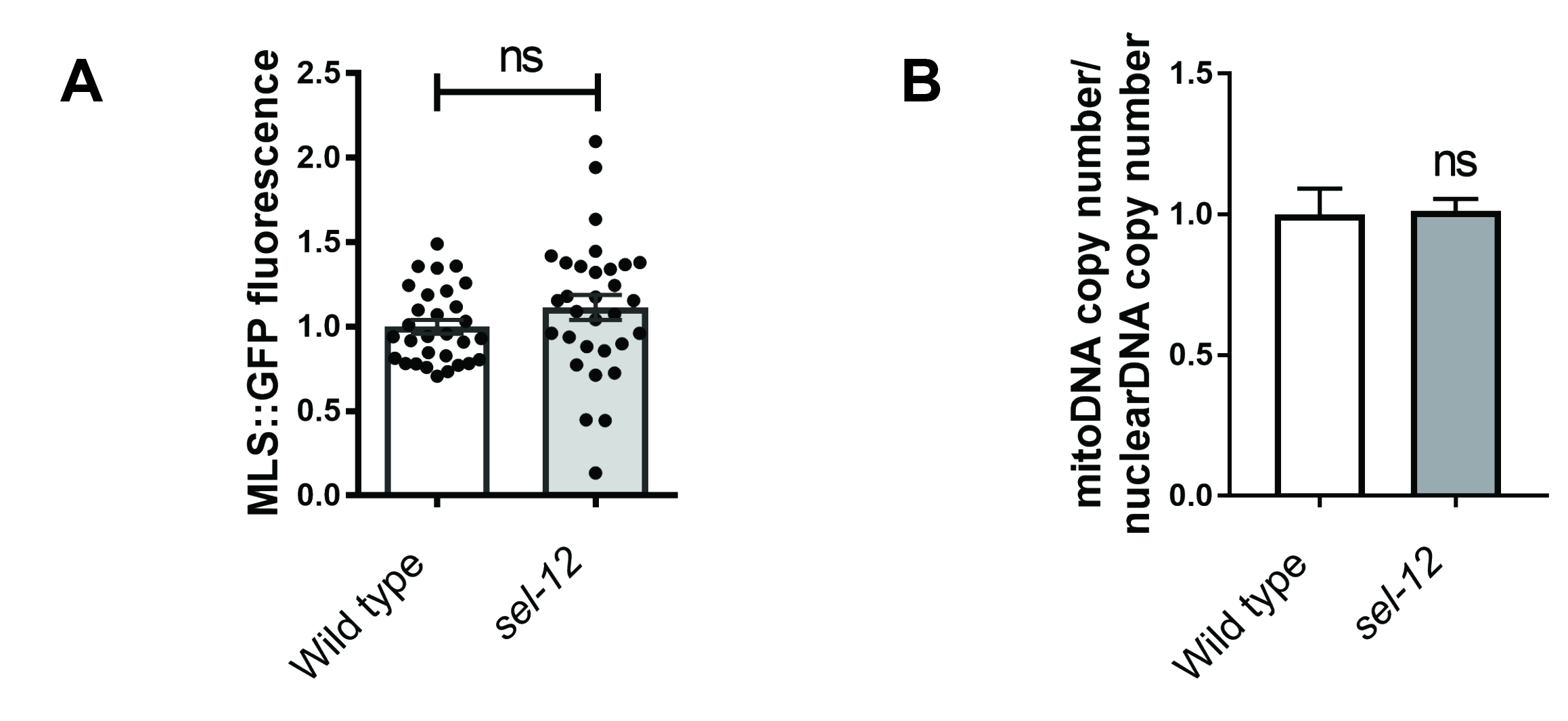


Figure S6

**Figure S1.** Animals with mTORC1 inhibited show increased lipidated LGG-1 levels. (A and B) Western blot of GFP::LGG-1 in day 1 adult worms (A) showing non-lipidated (upper band GFP::LGG-1-I) and lipidated (GFP::LGG-1-PE) band, and actin (loading control), and (B) quantification of GFP::LGG-1-PE/actin. n=3. ns P>0.05, *P<0.05, using two-tailed T-test. Error bars indicate mean +/- SEM. (C and F) Representative images of hypodermal seam cells (scale bar = 10 µm) (C) or muscle (scale bar = 10 µm) (F) expressing GFP::LGG-1/LC3 puncta (arrows) as a reporter for autophagosome formation. (D, E, and G) Quantification of GFP:LGG-1/LC3 puncta in seam cells (D and E) (n = 125 seam cells) or muscle (G) (n = 90 muscle cells). Error bars indicate mean +/- SEM. ns P>0.05, *P<0.05, ***P<0.001 using one-way ANOVA with Tukey’s multiple comparison test.

**Figure S2.** Metformin mTORC1 activation, restores oxygen consumption rate in *sel-12* mutants to normal levels, and improves soft touch behavior. (A and C) Western blot of p-RSKS-1 and actin (loading control), and (B and D) quantification of p-RSKS-1/actin. (E and F) Quantification of basal (E) and maximal (F) respiration in wild-type and *sel-12* animals with or without 50 μM metformin treatment. Assays were performed in triplicate with 20 worms per well. (E) Soft touch assay in wild-type or *sel-12* animals with or without metformin. Error bars indicate mean +/- SEM. ns P>0.05, *P<0.05, **P<0.01***P<0.001 using one-way ANOVA with Tukey’s multiple comparison test.

**Figure S3.** *mcu-1* mutants do not effect cytosolic calcium levels. (A) Representative images and (B) quantification of cytoplasmic calcium levels using transgenic animals expressing GCaMP3.35::SL2::mKate2 (*goeIs22*) in the mechanosensory neurons to measure relative fluorescence intensity. (n = 40). egl-19(gf) is a gain-of-function mutants in the *egl-19* gene, which encodes a voltage-gated calcium channel and causes elevated levels of cytosolic calcium. Error bars indicate mean +/- SEM. ns P>0.05, ***P<0.001 using one-way ANOVA with Tukey’s multiple comparison test.

**Figure S4.** Inhibition of mTORC1 restores the structural integrity of mechanosensory neurons in *sel-12* mutants. (A) Representative images of ALM/PLM axonal waves and breaks. (scale bar = 10 µm) (B-E) Average number of ALM and PLM axons displaying aberrant waves (B,D) and breaks (C,E). n ≥ 50. Error bars indicate mean +/-SEM. ns P>0.05, *P<0.05, **P<0.01, ***P<0.001 using one-way ANOVA with Tukey’s multiple comparison test. ns P>0.05.

**Figure S5.** mTORC2 activity and *hop-1* expression do not contribute to the neurodegeneration of *sel-12* mutants. (A) Average number of aberrant ectopic sprouts per ALM soma (n ≥ 30 animals). (B-D) Presence of aberrant lesions (B), waves (C), and breaks (D) per 10 ALM/PLM axons (n ≥ 60 axons). (E) Soft touch assay quantifying percentage response to anterior and posterior touch. (n ≥ 50 animals). (F) Real-time PCR of *hop-1* mRNA. *hop-1* expression was normalized to the housekeeping gene actin. *spr-4* mutants*,* which show elevated *hop-1* expression (Lakowski et al., 2003, Lu et al., 2014) were used as a positive control. (n=3 performed in triplicate). All error bars indicate SEM. ns P>0.05, *P<0.05, **P<0.01, ***P<0.001 using ANOVA with Tukey Test.

Figure S6. Mitochondrial content in sel-12 mutants is similar to wild-type animals. (A) Mitochondrial fluorescence intensity of wild-type and *sel-12* animals expressing a mitochondrial localization signal (MLS) tagged to GFP (*jsIs609*). (B) Mitochondrial DNA copy number normalized to nuclear DNA copy number in wild-type and *sel-12* animals. ns P>0.05, using two-tailed T-test. Error bars indicate mean +/- SEM.
